# Cross-species efficacy of AAV-mediated ARSA replacement for metachromatic leukodystrophy

**DOI:** 10.1172/JCI185001

**Published:** 2025-06-19

**Authors:** Shyam Ramachandran, Jeffery Ardinger, Jie Bu, MiAngela Ramos, Lilu Guo, Dhiman Ghosh, Mahmud Hossain, Shih-Ching Chou, Yao Chen, Erik Wischhof, Swathi Ayloo, Roger Trullo, Yuxia Luo, Jessica M. Hogestyn, Daniel M. DuBreuil, Emily Crosier, Johanna G. Flyer-Adams, Amy M. Richards, Michael Tsabar, Giorgio Gaglia, Shelley Nass, Bindu Nambiar, Denise Woodcock, Catherine O’Riordan, Qi Tang, Bradford Elmer, Bailin Zhang, Martin Goulet, Christian Mueller

**Affiliations:** 1 Genomic Medicine Unit, Sanofi, Waltham, Massachusetts, USA.; 2 Precision Medicine and Computational Biology, Sanofi, Cambridge, Massachusetts, USA.; 3 Artificial Intelligence and Deep Analytics, Sanofi, Bridgewater, New Jersey, USA.

**Keywords:** Genetics, Neuroscience, Gene therapy, Genetic diseases

## Abstract

Metachromatic leukodystrophy (MLD) is an autosomal recessive neurodegenerative disorder caused by mutations in the *arylsulfatase A* (*ARSA*) gene, resulting in lower sulfatase activity and the toxic accumulation of sulfatides in the central and peripheral nervous system. Children account for 70% of cases and become progressively disabled, with death occurring within 10 years of disease onset. Gene therapy approaches to restore ARSA expression via adeno-associated virus (AAV) vectors have been promising but hampered by limited brain biodistribution. We report the development of an engineered capsid, AAV.GMU01, demonstrating superior biodistribution and transgene expression in the central nervous system of nonhuman primates (NHPs). Next, we show that AAV.GMU01-*ARSA*–treated MLD mice exhibit persistent, normal levels of sulfatase activity and a concomitant reduction in toxic sulfatides. Treated mice also show a reduction in MLD-associated pathology and auditory dysfunction. Lastly, we demonstrate that treatment with AAV.GMU01-*ARSA* in NHPs is well tolerated and results in potentially therapeutic ARSA expression in the brain. In summary, we propose AAV.GMU01-*ARSA*–mediated gene replacement as a clinically viable approach to achieve broad and therapeutic levels of ARSA.

## Abstract 2

AAV-gene therapy for metachromatic leukodystrophy exhibits broad biodistribution, ARSA cross-correction and biologically meaningful expression in NHPs.

## Graphical abstract

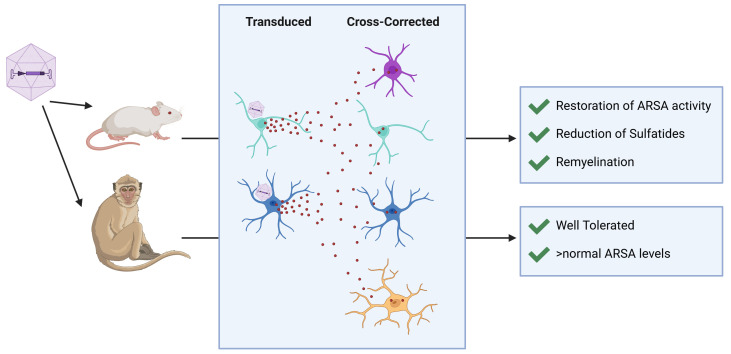

## Introduction

Metachromatic leukodystrophy (MLD) is a progressive, autosomal recessive neurodegenerative disorder caused by mutations in the *arylsulfatase A* (*ARSA*) gene, which result in a deficiency of the lysosomal enzyme ARSA and the pathological accumulation of sulfatides in cells (1–3). Over 200 mutations in the *ARSA* gene have been linked to MLD (4), leading to the buildup of 3-*O*-sulfogalactosyl ceramides (sulfatides) and their deacylated form, lyso-sulfatide (lyso-ST), a distinct marker of disease pathology (5, 6). Sulfatide accumulation in oligodendrocytes and Schwann cells (5, 7) drives demyelination in both the CNS and PNS (8–10), while short-chain fatty acid sulfatides in neurons and astrocytes contribute to neuronal dysfunction, cell loss, and progressive neurodegeneration (8, 11–15). In the CNS, this disruption destabilizes myelin, impairing oligodendrocyte function and leading to widespread demyelination in regions such as the basal ganglia, cerebellum, and spinal cord, resulting in cognitive decline, motor dysfunction, spastic tetraparesis, ataxia, spasms, and seizures (13, 16, 17). Similarly, PNS demyelination causes peripheral neuropathy, muscle weakness, and sensory deficits (13). Sulfatide accumulation also triggers calcium buildup and cellular stress (18), further exacerbating myelin instability and neurodegeneration (19). While non-neural tissues, such as the kidneys and gallbladder, are also affected (20), clinical symptoms are primarily concentrated in the nervous system, making the restoration of ARSA activity in both the CNS and PNS the primary therapeutic goal to mitigate the toxic effects of sulfatide accumulation.

MLD manifests in 3 primary forms, late infantile, juvenile, and adult, delineated by the age of onset. While the initial symptoms vary, all patients experience progressive and severe disability, regression of motor skills, gait difficulties, ataxia, and weakness. As the disease progresses, patients experience dysphagia, feeding intolerance, seizures, hypotonia, and peripheral neuropathy. Those with late infantile MLD have a mean life expectancy of around 4 years (21). Currently, atidarsagene autotemcel (Libmeldy/Lenmeldy, Orchard Therapeutics), an autologous hematopoietic stem cell therapy, is the sole approved disease-modifying therapy for MLD (17). However, its approval is limited to presymptomatic late infantile or early-juvenile patients without neurological impairment (22). Other treatment options are limited to palliative care, symptom alleviation, tube feeding, and psychological support. Thus, a considerable unmet need still exists for MLD patients.

Adeno-associated viruses (AAVs) deliver sustained expression of their transgene cargo, and AAV-mediated gene therapy has shown success in treating neurological disorders (23–26). Over 300 patients across 14 types of neurological disorders have undergone AAV infusions, showing good tolerance and safety (27–30). Here, we report the development of an AAV-mediated gene therapy for MLD. This therapy comprises an engineered AAV9 variant, AAV.GMU01, with enhanced CNS targeting in nonhuman primates (NHPs) and a WT human *ARSA* cDNA. We show that delivery of AAV.GMU01-*ARSA* into the CNS of *Arsa*-KO mice results in a reduction of toxic sulfatides and phenotypic reversal. In NHPs, we demonstrate broad biodistribution and a CNS-wide therapeutic footprint — enabled by ARSA protein cross-correction — following delivery via a minimally invasive and clinically feasible administration route. Finally, we establish in NHPs that this treatment delivers potentially therapeutic doses.

## Results

### AAV.GMU01 yields superior biodistribution compared with AAV.rh10 in cynomolgus NHPs

MLD affects all regions of the nervous system, and an effective therapy requires widespread distribution of ARSA protein. AAV.rh10, a naturally occurring capsid, efficiently targets the CNS in both rodents and NHPs (31, 32). However, despite promising preclinical results (33–35), a clinical trial employing intraparenchymal delivery of AAV.rh10 encoding *ARSA* did not achieve therapeutic endpoints (36). We aimed to identify a capsid that could yield enhanced biodistribution and superior transgene expression when delivered via a minimally invasive, intra-CSF route of administration (RoA).

**Figure 1:**
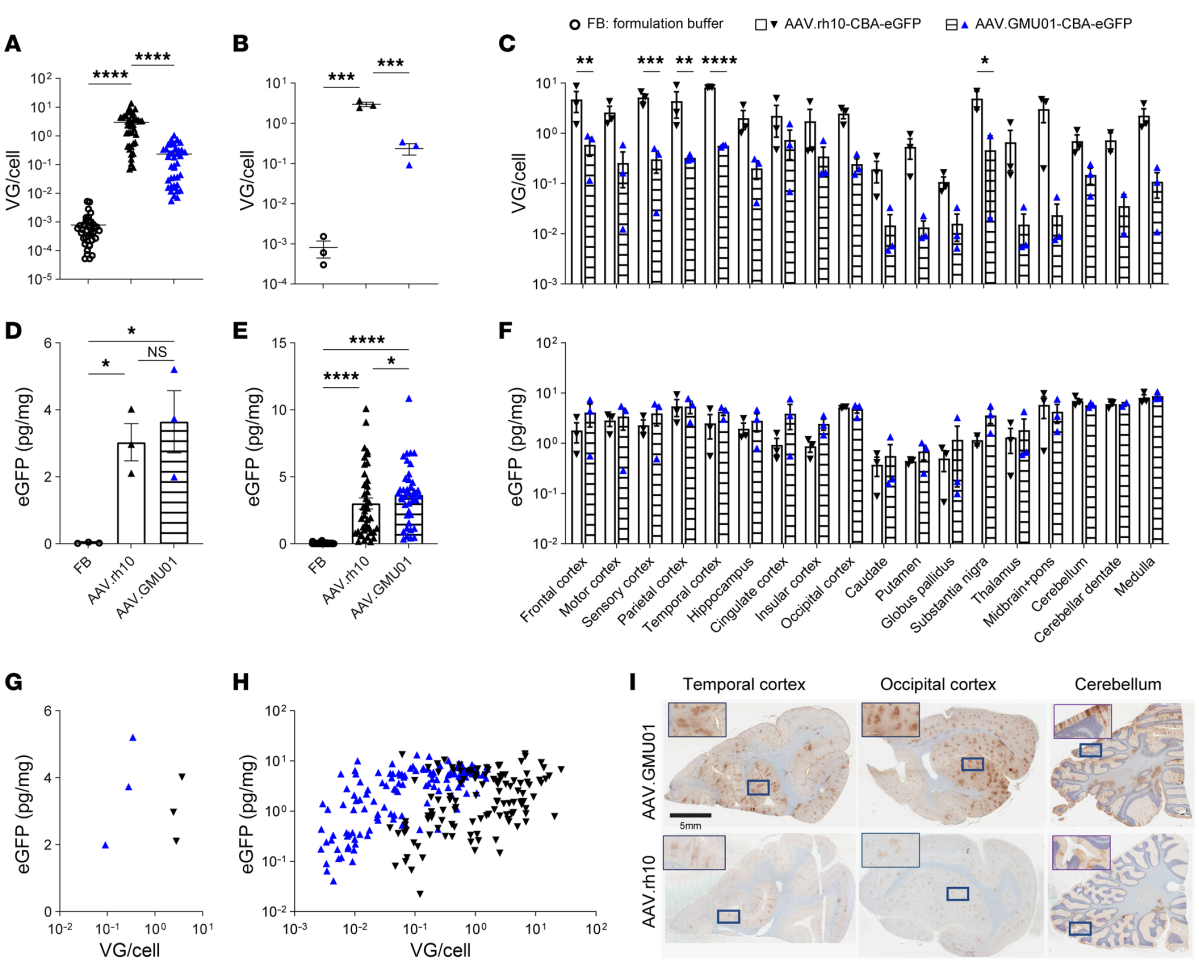
The engineered capsid AAV.GMU01 shows higher transgene expression in the brain of NHPs compared with AAV.rh10. Cynomolgus monkeys (male, Mauritius, 2 years old, 2–3 kg) seronegative for AAV.rh10 and AAV.GMU01 were dosed by intrathecal delivery at the cervical level 1–2 junction using a ported intrathecal catheter inserted at the lumbar region. Animals were dosed in the Trendelenburg position. One dose of AAV.GMU01 or rh10-CBA-eGFP was administrated at 2.75e13 VG/NHP (3.65e11 VG/g brain weight). At 16–29 days after dose, animals were euthanized. (**A**–**H**) Forty-one tissue biopsy punches from 19 different gray matter brain regions were taken. AAV VG copies (**A**–**C**) were measured in brain biopsy punches by bovine growth hormone-digital polymerase chain reaction and normalized to *TUBB1* gene intron to obtain VG copies per cell, presented by animal (**A**) or by punch (**B** and **C**). eGFP expression (**D**–**F**) in the same regions was measured by ELISA, presented by animal (**D**) or by punch (**E** and **F**). (**G** and **H**) Correlation of vector exposure to eGFP expression in brain, presented by animal (**G**) or by punch (**H**). (**I**) Representative images depicting distinct brain regions from AAV.GMU01- and AAV.rh10-treated NHPs stained for eGFP. Scale bar: 5 mm. Data are shown as the mean ± SEM. Two-way ANOVA with Tukey’s or Šidák’s (**B** and **D**) multiple-comparison test. *P < 0.05; **P < 0.01; ***P < 0.001; ****P < 0.0001.

Screening of a 7–amino acid peptide insert library based on the AAV9 capsid in rhesus macaques via intracerebroventricular (ICV) injection identified AAV.GMU01, with significantly enhanced vector biodistribution and transgene expression in critical CNS regions compared with the parental AAV9 capsid (37). To further evaluate its therapeutic potential, we compared the biodistribution and transgene expression of an eGFP-encoding AAV.GMU01 to AAV.rh10 following intrathecal delivery (Supplemental Table 1; supplemental material available online with this article; https://doi.org/10.1172/JCI185001DS1). We administered AAV.GMU01-CBA-*eGFP* or AAV.rh10-CBA-*eGFP* to cynomolgus monkeys at the cervical level 1–2 junction at a dose of 2.75e13 vector genomes (VGs) per animal; 16–29 days later, we evaluated vector exposure and eGFP expression. Despite displaying approximately 10-fold lower vector exposure across 41 gray matter punches (representing 16 brain regions) than AAV.rh10-treated NHPs (Figure 1, A–C), AAV.GMU01 exhibited improved eGFP expression (Figure 1, D–F). To visualize this difference, we plotted the correlation between eGFP expression and VGs, either by animal or by each of the 41 gray matter punches. This plot demonstrated a leftward shift in AAV.GMU01 correlation, relative to AAV.rh10, which signified higher expression at lower brain VG exposure (Figure 1, G and H). Immunohistochemistry against eGFP confirmed robust expression in AAV.GMU01-treated NHPs (Figure 1I). AAV.GMU01-treated NHPs also exhibited lower VGs in the spinal cord, dorsal root ganglia (DRGs), liver, heart, lung, and kidney with only the spleen showing higher exposure (Supplemental Figure 1A). Despite this, eGFP levels in spinal cord and DRGs were higher than or similar to those achieved by AAV.rh10 (Supplemental Figure 1B). These results indicate that AAV.GMU01 achieves widespread CNS biodistribution and transgene expression at a significantly lower AAV vector exposure than AAV.rh10.

### Comparison and selection of CSF delivery route

While the i.v. and intraparenchymal routes of administration can achieve AAV distribution to the CNS, both have considerable drawbacks. IV delivery requires high doses that present the risk of toxicity in non–target tissue. Intra-CSF delivery achieves more widespread biodistribution to the CNS at lower doses than i.v. administration (38–41), and low concentrations of IgG in the CSF make intra-CSF delivery less prone to risks related to preexisting neutralizing antibodies (42). Meanwhile, intraparenchymal delivery often results in nonhomogenous distribution (43). Therefore, intra-CSF is the preferred RoA for CNS-targeted therapies. To establish an optimal intra-CSF route of delivery, we examined the biodistribution of AAV.GMU01 delivered by 1 of 2 different routes: (a) bilateral ICV and (b) direct injection to the cisterna magna (ICM). We chose to evaluate the ICM route both because it is a clinically feasible and minimally invasive method for direct access to nervous system tissues (38–41, 44) and because it achieves better biodistribution to the CNS than intrathecal-lumbar administration (40, 43, 45).

**Figure 2:**
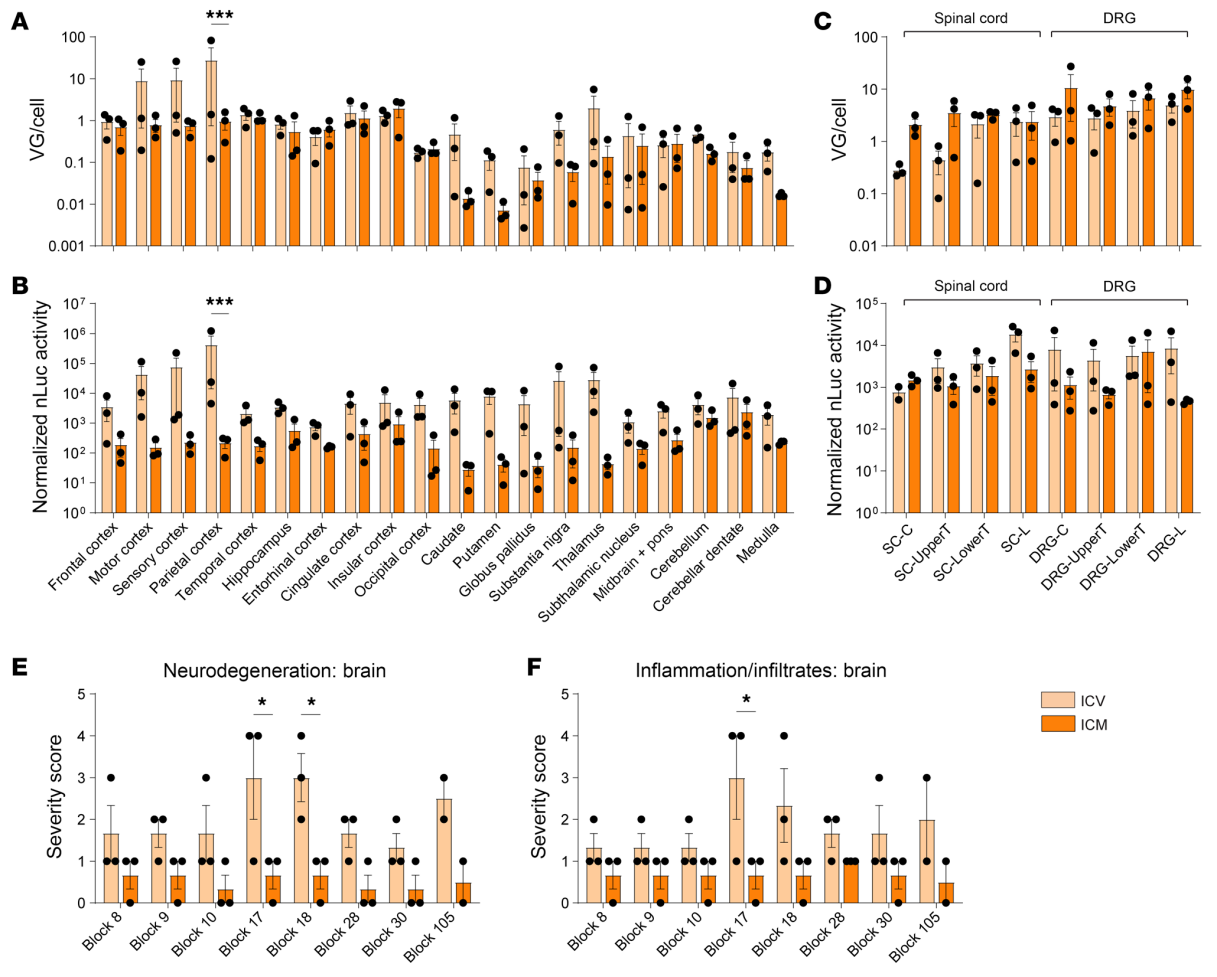
AAV.GMU01 shows widespread vector biodistribution and transgene activity throughout brain, spinal cord, and DRGs. Cynomolgus monkeys (male, Vietnam, 2–3 years old, 2–3 kg) seronegative for AAV.GMU01 were dosed with AAV.GMU01-CBA-nLuc-mCherry at 2.0e13 VG/NHP (2.75e11 VG/g brain weight) either by bilateral ICV injection or direct ICM. Four weeks after dose, animals were euthanized, and brain, spinal cord, and DRG tissues were flash-frozen. Tissue biopsy punches corresponding to the indicated brain regions and 4 spinal cord levels were assessed for VG exposure (**A** and **C**) by dPCR. nLuc activity (**B** and **D**) was assessed using the Nano-Glo luciferase assay and normalized to total protein measured by bicinchoninic acid assay. (**E** and **F**) Histopathological findings in brain, spinal cord, and DRG; each data point represents the maximum severity of findings scored on 1–2 sections per animal. Severity scores refer to findings graded as 0 = no findings, 1 = minimal, 2 = mild, 3 = moderate, 4 = marked, and 5 = severe. Block 8: frontal cortex, striatum; Block 9: insular cortex, temporal cortex; Block 10: motor cortex, striatum; Block 17: thalamus, insular cortex; Block 18: hippocampus, entorhinal cortex; Block 28: parietal cortex; Block 30: cerebellum, medulla, Block 105: midbrain, pons. Data are shown as the mean ± SEM; 1- and 2-way ANOVA with Šidák’s multiple-comparison test. *P < 0.05; ***P < 0.001.

Cynomolgus NHPs were administered AAV.GMU01-CBA-nLuc-mCherry at a dose of 2.0e13 VGs per animal (Supplemental Table 2). After 4 weeks, we collected brain, spinal cord, DRG, and peripheral tissues and analyzed VGs and luciferase activity. Histopathological analysis and quantification of neurofilament light chain (Nf-L) were also conducted to assess the relative tolerability of each RoA. In 20 brain regions, both administration routes demonstrated a broad distribution of VGs (Figure 2A). Cortical regions exhibited transduction levels of approximately 1 VG/cell, with ICV delivery showing a trend toward increased transduction in subcortical structures. ICV delivery demonstrated significantly increased nanoluciferase (nLuc) activity across the brain, particularly in cortical and subcortical regions (Figure 2B). Comparable vector biodistribution and transgene activity were observed in the spinal cord, DRGs (Figure 2, C and D), and other peripheral organs (Supplemental Figure 2, A and B). Neither RoA resulted in behavioral signs, and both caused a similar increase in Nf-L in the CSF (Supplemental Figure 2C). However, minimal to marked inflammatory and degenerative histopathological findings were noted in the brain of ICV-treated NHPs, whereas the ICM group exhibited only minimal findings (Figure 2, E and F). In the spinal cord and DRGs, minimal to moderate inflammatory and degenerative findings were observed in all groups (Supplemental Figure 2, D–G), an almost universal subclinical finding after AAV gene therapy (46). To evaluate cellular tropism in the NHP brain, we conducted an immunofluorescence codetection assay, demonstrating transduction to neurons, astrocytes, microglia, and oligodendrocytes (Supplemental Figure 3). Additional studies using single-nucleus RNA sequencing would be needed to further explore cell-type–specific transduction across NHP brain tissues. In summary, we concluded that ICM delivery of AAV.GMU01 is well tolerated and results in widespread transduction in the CNS.

### AAV.GMU01-ARSA administration is well tolerated, long lasting, and effective in MLD mice

To evaluate the short- and long-term efficacy of ARSA replacement, we conducted 4 pharmacology studies in MLD mice using AAV.GMU01-*ARSA*, targeting different stages of neuropathological disease progression (Supplemental Figure 4A). The MLD mouse model (referred to as *Arsa*-KO) was generated by Sanofi (described in supplemental information) and exhibits characteristic sulfatide accumulation in the CNS and visceral organs, evidence of modest gliosis and microglial activation, and an auditory deficit phenotype similar to the original Gieselmann model (47, 48). We selected the ICV administration route for its reproducibility and efficient AAV vector delivery into the CSF of mice. Additionally, in WT mice treated with AAV.GMU01-eGFP at a dose of 1.6e11 VGs per mouse, single-cell RNA sequencing revealed transduction of approximately 12% of cells in the forebrain and 3% in the hindbrain (Supplemental Figure 4, B–E).

#### Long-term pharmacology and efficacy.

**Figure 3:**
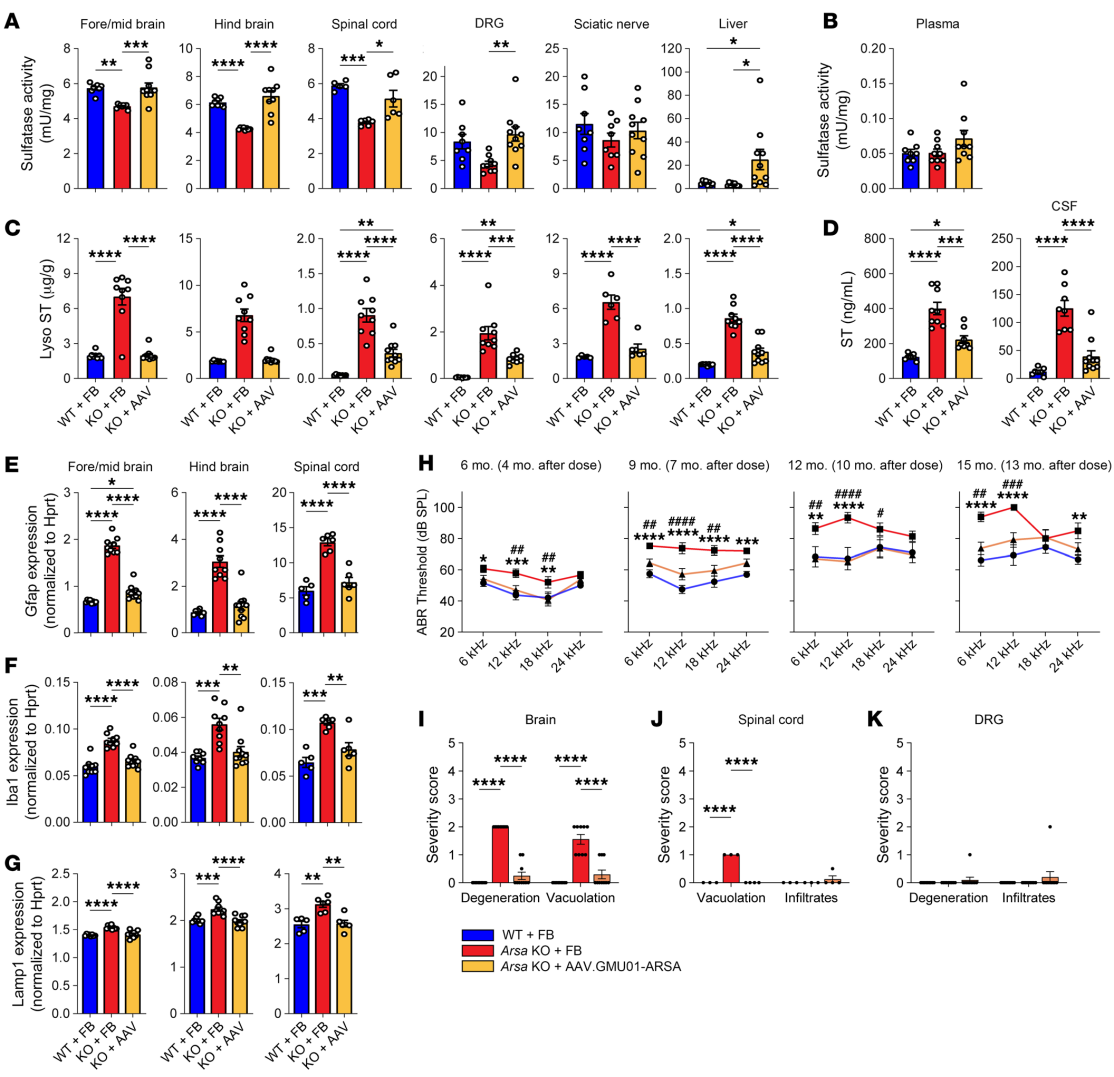
Phenotypic reversal in *Arsa*-KO mice treated with AAV.GMU01-*ARSA*. Pre-neuronopathic *Arsa*-KO mice and age-matched control animals were dosed with AAV.GMU01-*ARSA* at 1.6e11 VG/mouse (3.3e11 VG/g brain weight). Thirteen months after dose, *Arsa*-KO mice were euthanized, and brain, spinal cord, DRG, sciatic nerve, liver, plasma, and CSF samples were collected. (**A** and **B**) ARSA-mediated sulfatase activity was measured using the sulfatase activity assay; data normalized to total protein measured by bicinchoninic acid (BCA) assay. (**C** and **D**) Sulfatide levels were measured using liquid chromatography–mass spectrometry. Data normalized to tissue weight: converted from ng/mL (50 μL) to μg/g (ng ST/μg total protein for DRG and sciatic nerve). For fluids, data are presented as ng/mL (CSF). (**E**–**G**) RT-dPCR was performed to quantify *Gfap*, *Aif1* (gene for Iba1), and *Lamp1* levels, normalized to mouse *Hprt* gene. Each data point represents a single animal. Data are shown as the mean ± SEM; 1-way ANOVA with Tukey’s multiple-comparison test. (**H**) At 4, 7, 10, and 13 months after dose, ABR measurements were recorded via electrodes placed on the scalp of an anesthetized animal. Data are shown as the mean ± SEM; 2-way ANOVA with Tukey’s multiple-comparison test. ^#^denotes “KO+FB” versus “KO+AAV” group; *denotes “WT+FB” versus “KO+FB” group. (**I**–**K**) Histopathological findings in brain, spinal cord, and DRG; each data point represents the maximum severity of findings scored on 1–2 sections per animal. Severity scores refer to findings graded as 0 = no findings, 1 = minimal, 2 = mild, 3 = moderate, 4 = marked, and 5 = severe. Data are shown as the mean ± SEM. *^/#^*P* < 0.05; **^/##^*P* < 0.01; ***^/###^*P* < 0.001; ****^/####^*P* < 0.0001. FB, formulation buffer; ST, sulfatide.

To assess efficacy of long-term ARSA replacement, we administered AAV.GMU01-*ARSA* at a dose of 1.6e11 VGs per mouse to pre-neuropathic *Arsa*-KO mice and monitored them for 13 months; formulation buffer–treated WT and Arsa-KO mice served as controls (study design in Supplemental Table 3). AAV.GMU01 VGs and *ARSA* mRNA expression were measured in brain and spinal cord samples (Supplemental Figure 5, A and B), and sulfatase activity was measured in several brain regions as well as spinal cord, DRGs, sciatic nerve, liver, and plasma. AAV.GMU01-*ARSA* treatment restored sulfatase activity to levels equivalent to WT levels in the brain, spinal cord, DRGs, and sciatic nerve (Figure 3A). In the liver and plasma, sulfatase activity was higher in treated mice compared with WT (Figure 3, A and B). We observed a corresponding reduction in sulfatide deposition, which is the primary driver of toxicity in patients. Treatment normalized lyso-ST, C16, C18, and total ST levels in the brain and spinal cord and significantly reduced lyso-ST, C16, C18, and total ST levels in DRGs and the sciatic nerve (Figure 3C and Supplemental Figure 6, A–C). MLD patients exhibit an increase in sulfatides in biological fluids (5), making them a critical biomarker readout in the clinic. We observed a substantial reduction in total sulfatides in plasma and cerebrospinal fluid of treated mice (Figure 3D). In the brain and spinal cord, the decrease in sulfatide levels coincided with a significant reduction in neuroinflammatory marker levels (*Gfap* and *Aif1*) and in the expression of *Lamp1*, a marker of immune cell activation and degradative lysosomes (Figure 3, E–G). Furthermore, compared with buffer-treated *Arsa*-KO mice, AAV.GMU01-*ARSA* treated mice displayed a significant reduction in plasma Nf-L levels (Supplemental Figure 6D).

Hearing impairment is common in MLD patients. The Auditory Brainstem Response (ABR) test provides functional insights into both the inner ear (cochlea) and central hearing pathways. Mice and humans perceive multiharmonic communication sounds similarly (49), with mice having the best hearing range between 12 and 20 kHz. While mice perceive high-frequency sounds (12–20 kHz) better than low-frequency sounds (3–12 kHz), like humans, they respond better to low-frequency sounds (49). *Arsa*-KO mice exhibit a progressive decline in sound perception over time, with the largest change in ABR observed at lower frequencies (Figure 3H). Treatment with AAV.GMU01-*ARSA* largely prevented the loss of hearing in *Arsa*-KO mice (Figure 3H), with the difference between groups appearing as early as 4 months after dosing (earliest time point assessed), indicating the potentially rapid physiological and functional benefits of AAV-mediated ARSA gene replacement.

Consistent with other models, *Arsa*-KO mice displayed histopathological changes, including vacuolation and neurodegeneration, chiefly in the brainstem and/or cerebellum (Figure 3I). Treated mice exhibited a notable reduction in both the severity and incidence of these histological changes (Figure 3I). Additionally, all untreated *Arsa*-KO mice exhibited vacuolation in the spinal cord, which was absent in both WT and treated mice (Figure 3J). Histopathological alterations in the DRGs occurred in only 1 treated mouse (Figure 3K).

#### Longitudinal pharmacology.

**Figure 4:**
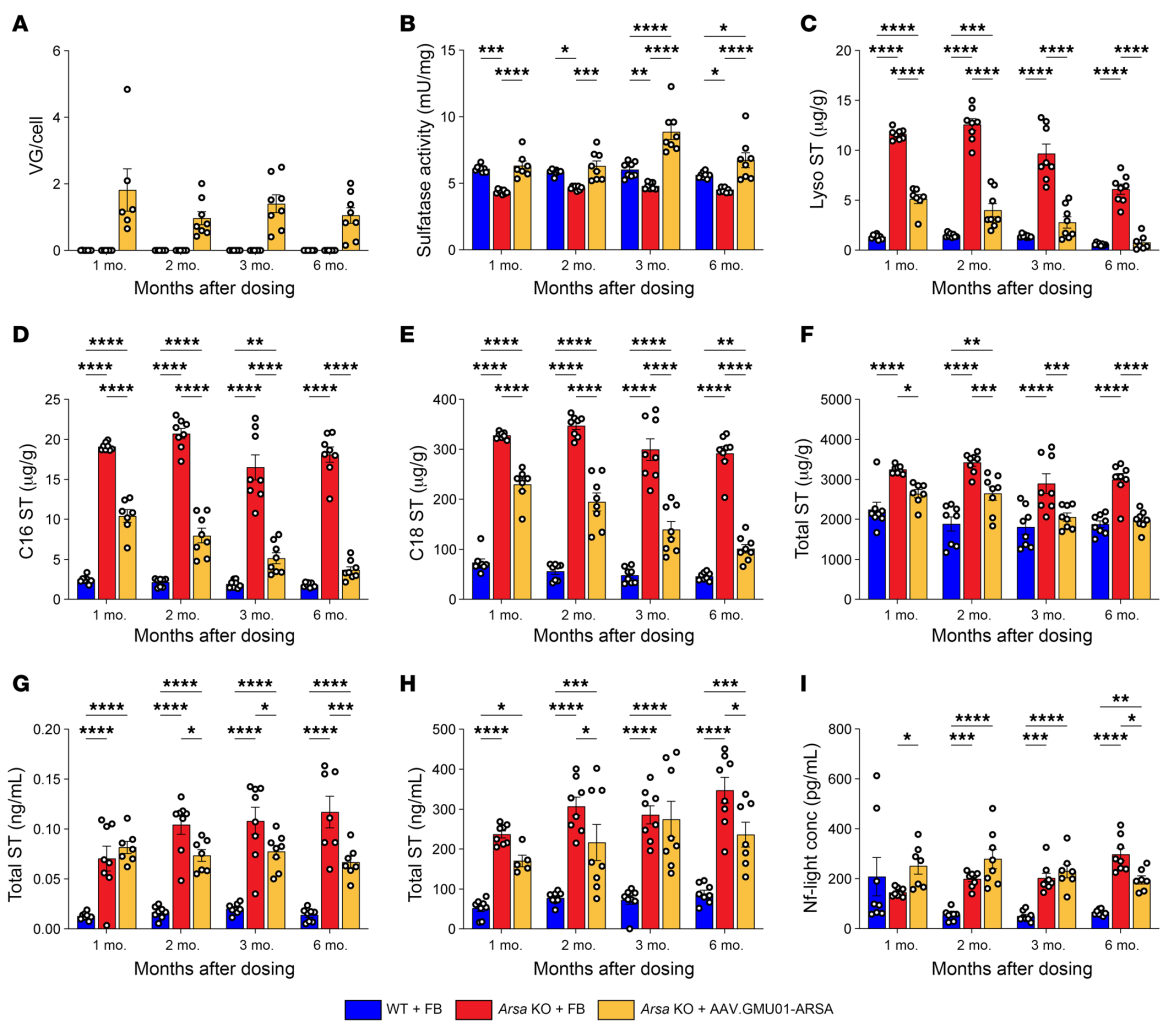
ARSA expression and function are persistent over time. Early-neuronopathic *Arsa*-KO mice (6 months at dosing) and age-matched control animals were dosed with AAV.GMU01-*ARSA* at 5e10 VG/mouse (1.0e11 VG/g brain weight). At 1, 2, 3, and 6 months after dose, *Arsa*-KO mice were euthanized and samples collected. (**A**–**F**) Brain (forebrain + midbrain) samples. (**A**) Vector exposure by bovine growth hormone-digital polymerase chain reaction normalized to the *Rab1a* gene (intronic region). (**B**) ARSA-mediated sulfatase activity was measured using the sulfatase activity assay kit, and data were normalized to total protein measured by bicinchoninic acid assay. (**C**) Lyso-ST, (**D**) C16-sulfatide isoform, (**E**) C18-sulfatide isoform, and (**F**) total sulfatide levels were measured using liquid chromatography–mass spectrometry. Data were normalized to tissue weight: converted from ng/mL (50 μL) to μg/g. Total sulfatide levels were also measured in (**G**) CSF and (**H**) plasma samples. (**I**) Plasma samples were collected at necropsy and assayed using the Simoa platform (Quanterix) to quantify Nf-L. Data are shown as the mean ± SEM. Two-way ANOVA with Tukey’s multiple-comparison test. *P < 0.05; **P < 0.01; ***P < 0.001; ****P < 0.0001. FB, formulation buffer; ST, sulfatide.

This study aimed to assess target engagement and reversibility of MLD-associated pathological and biochemical traits in the *Arsa*-KO model over a 6-month period. Six-month-old early-neuronopathic *Arsa*-KO mice underwent bilateral ICV injections of AAV.GMU01-ARSA at a dose of 5.0e10 VGs per mouse. Control groups, including age-matched WT and *Arsa*-KO mice, received doses of formulation buffer (study design in Supplemental Table 4). Animals were monitored for 6 months, with assessments of VGs, sulfatase activity, and sulfatide levels performed at 1, 2, 3, and 6 months after dose. AAV.GMU01-*ARSA* VG exposure in the brain (forebrain + midbrain) remained consistent over the 6-month period (Figure 4A). Sulfatase activity was restored to levels equivalent to WT animals (Figure 4B) and reached its peak by 1 month after dose. We observed significant reductions in sulfatide deposition, including in lyso-ST, C16, C18, and total ST, at all time points (Figure 4, C–F). Clearance of sulfatides was progressive, returning to normal by 6 months after dose. Due to low vector exposure in the hindbrain and spinal cord (Supplemental Figure 7, A and G), we measured only a minimal increase in sulfatase activity (Supplemental Figure 7, B and H); however, lyso-ST, C16, C18, and total ST were all reduced in both tissues (Supplemental Figure 7, C–E and I–L), suggesting ARSA protein distribution to the hindbrain and spinal cord. Furthermore, AAV.GMU01-*ARSA* treatment significantly reduced total ST levels in cerebrospinal fluid and plasma (Figure 4, G and H). AAV administration resulted in a marginal increase in plasma Nf-L levels, which resolved over time, and by 6 months after dose, plasma Nf-L levels were significantly decreased (Figure 4I).

Notably, a reduction in the incidence and severity of neuronal and neuropil changes was evident in the brains of AAV.GMU01-*ARSA* treated *Arsa*-KO mice, as early as 2 months after dose (Supplemental Figure 8). In the brains of some AAV.GMU01-*ARSA* treated mice, perivascular infiltrates of mononuclear cells with minimal severity were observed, usually near the hippocampus (Supplemental Figure 8). Additionally, increased cellularity of glial cells and/or mononuclear cells, typically of minimal or occasionally mild severity, was also present in the DRGs of certain AAV.GMU01-*ARSA* treated mice (Supplemental Figure 8). No test article–related histopathological changes were detected in the spinal cord (Supplemental Figure 8). Histological changes in the sciatic nerve were observed in most *Arsa*-KO mice, with the incidence and severity occasionally increased to minimal or mild in AAV.GMU01-*ARSA* treated *Arsa*-KO mice (Supplemental Figure 8).

#### Dose-dependent pharmacology.

**Figure 5:**
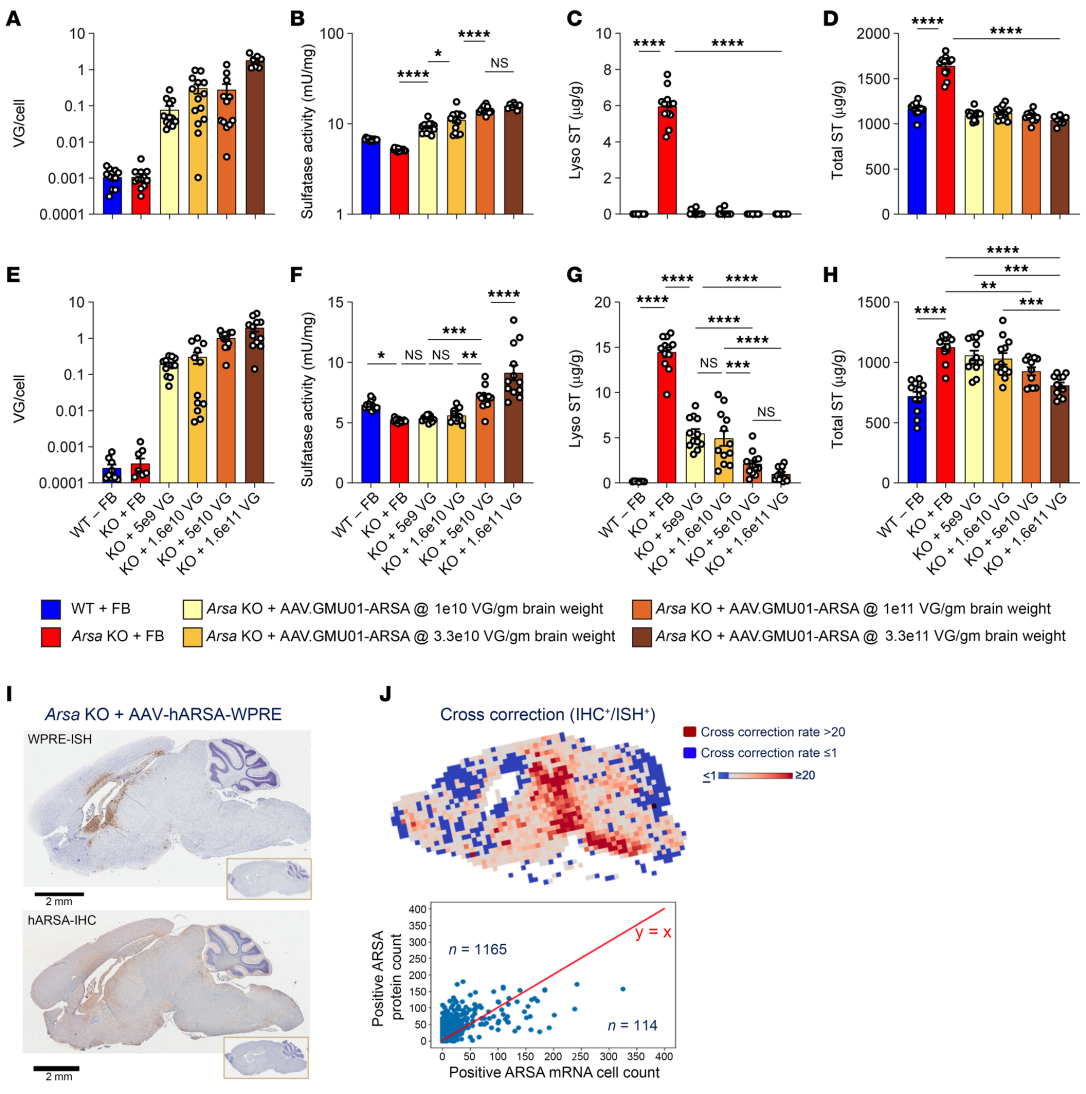
AAV-*ARSA* treatment results in dose-dependent sulfatide clearance in *Arsa*-KO mice with evidence of robust cross-correction. (**A**–**H**) Brain (forebrain + midbrain) samples. (**A**–**D**) Neonatal *Arsa*-KO mice (P0 at dosing) and age-matched control animals were dosed with AAV.GMU01-*ARSA* at noted doses. Six months after dose, mice were euthanized and samples collected. (**A**) Vector exposure by bovine growth hormone-digital polymerase chain reaction (bGH-dPCR) normalized to the *Rab1a* gene (intronic region). (**B**) ARSA-mediated sulfatase activity was measured using the sulfatase activity assay kit and data normalized to total protein measured by bicinchoninic acid (BCA) assay. (**C**) Lyso-ST and (**D**) total sulfatide levels were measured using liquid chromatography–mass spectrometry (LC-MS). Data normalized to tissue weight: converted from ng/mL (50 μL) to μg/g. (**E**–**H**) Early-neuronopathic *Arsa*-KO mice (6 months at dosing) and age-matched control animals were dosed with AAV.GMU01-*ARSA* at noted doses. Three months after dose, mice were euthanized and samples collected. gm, gram. (**E**) Vector exposure by bGH-dPCR normalized to the *Rab1a* gene (intronic region). (**F**) ARSA-mediated sulfatase activity was measured using the sulfatase activity assay kit and data normalized to total protein measured by BCA assay. (**G**) Lyso-ST and (**H**) total sulfatide levels were measured using LC-MS. Data normalized to tissue weight: converted from ng/mL (50 μL) to μg/g. Data are shown as the mean ± SEM. Two-way ANOVA with Tukey’s multiple-comparison test. *P < 0.05; **P < 0.01; ***P < 0.001; ****P < 0.0001. FB, formulation buffer; ST, sulfatide. (**I** and **J**) AAV.rh10-ARSA-WPRE–treated *Arsa*-KO mice showing evidence of ARSA protein cross-correction. Late-stage (13 month) *Arsa*-KO mice were dosed with AAV.rh10-CBA-ARSA-WPRE. Three months after dose, ARSA mRNA ISH and ARSA protein IHC were performed on matched sagittal brain hemisections. The sections were imaged and analyzed for signal overlay. (**I**) Representative sections from mouse brain stained for *ARSA* mRNA (ISH) and ARSA protein (IHC) with DAPI staining for nuclei (inset: staining from buffer-treated *Arsa*-KO mice). Scale bars: 2 mm. (**J**) Cross-correction factor (ratio of IHC^+^ cells to ISH^+^ cells) is represented as a heatmap with highly cross-corrected tiles shown in shades of red. ISH+ cell count versus IHC^+^ cell count from each tile is plotted as a scatterplot with *y* = *x* line shown in red. Tiles above the *y* = *x* line indicate cross-corrected cells.

To evaluate dose-dependent ARSA expression and its impact on efficacy (sulfatide clearance), we ran 2 studies, 1 in neonatal (dosing at P0) and another in early-neuronopathic (dosing at 6 months of age) *Arsa*-KO mice. Mice underwent bilateral ICV injections of AAV.GMU01-*ARSA* at 4 increasing doses ranging from 1e10 to 3.3e11 VG/g brain weight. Control groups, including age-matched WT and *Arsa*-KO mice, received formulation buffer (study design in Supplemental Table 5). Six months after dose, animals treated neonatally showed dose-dependent vector biodistribution (Figure 5A) and a robust increase in sulfatase activity in the brain (forebrain + midbrain), with levels surpassing those of WT mice at all doses (Figure 5B). We also observed a concomitant decrease in sulfatide deposits in the brain (Figure 5, C and D, and Supplemental Figure 9, A and B). Similarly, 3 months after dose, early-neuronopathic mice showed a dose-dependent vector biodistribution (Figure 5E) and a robust increase in brain (forebrain + midbrain) sulfatase activity at the top 2 doses (Figure 5F), with a significant decrease in sulfatide deposits in the brain across all doses (Figure 5, G and H, and Supplemental Figure 9, C and D).

Taken together, these data demonstrate that, in *Arsa*-KO mice, AAV.GMU01-*ARSA*–mediated gene replacement is well tolerated and rectifies MLD-associated phenotypes, validating our therapeutic approach.

### ARSA protein cross-correction generates a large therapeutic footprint

As mentioned above, in certain tissues, we observed a discrepancy between sulfatase activity and sulfatide levels with a minimal increase in activity generating a larger than expected reduction in sulfatides. While the relative sensitivity of the 2 assays likely explains much of this discrepancy, in some tissues (hindbrain and spinal cord), very low vector exposure leads to robust sulfatide clearance. ARSA enzyme is naturally secreted into the extracellular matrix, where it is taken up by neighboring and distant cells via the mannose 6-phosphate receptor (50–52). Our data suggest that the recombinant human protein can leverage normal ARSA secretion and reuptake mechanisms facilitating enzyme spread to secondary, nontransduced cells, resulting in cross-correction in a broad range of cells (50–52), including oligodendrocytes (13, 53) and microglia (54), which are not efficiently transduced in vivo by AAV vectors.

To measure the extent of cross-correction, 13-month-old *Arsa*-KO mice underwent bilateral ICV injections of AAV.rh10-*ARSA* at a dose of 1.6e11 VGs per mouse. Three months after dose, consecutive 5 mm brain sections were processed for *ARSA* mRNA ISH and ARSA protein IHC (Figure 5I), imaged, and registered, and the ratio of cells containing ARSA protein to cells containing the VG was determined (Figure 5J). We observed robust ARSA protein biodistribution throughout the brain, with more than 84% of brain by area showing evidence of cross-correction (Figure 5J and Supplemental Figure 10). In neonatal *Arsa*-KO mice dosed bilaterally (ICV) at P0 with AAV.GMU01-*ARSA* at 3.3e11 VG/g brain weight, we performed an immunofluorescence codetection assay to evaluate cross-correction. These animals demonstrated a robust increase in sulfatase activity and a concomitant decrease in sulfatide deposits in the brain (Figure 5, B–D, and Supplemental Figure 9, A and B). Additionally, cross-correction was evident in neurons, astrocytes, and microglia (Supplemental Figure 11), highlighting the widespread therapeutic potential of the vector.

### AAV.GMU01-ARSA treatment is well tolerated in NHPs and results in widespread ARSA expression in CNS

**Figure 6:**
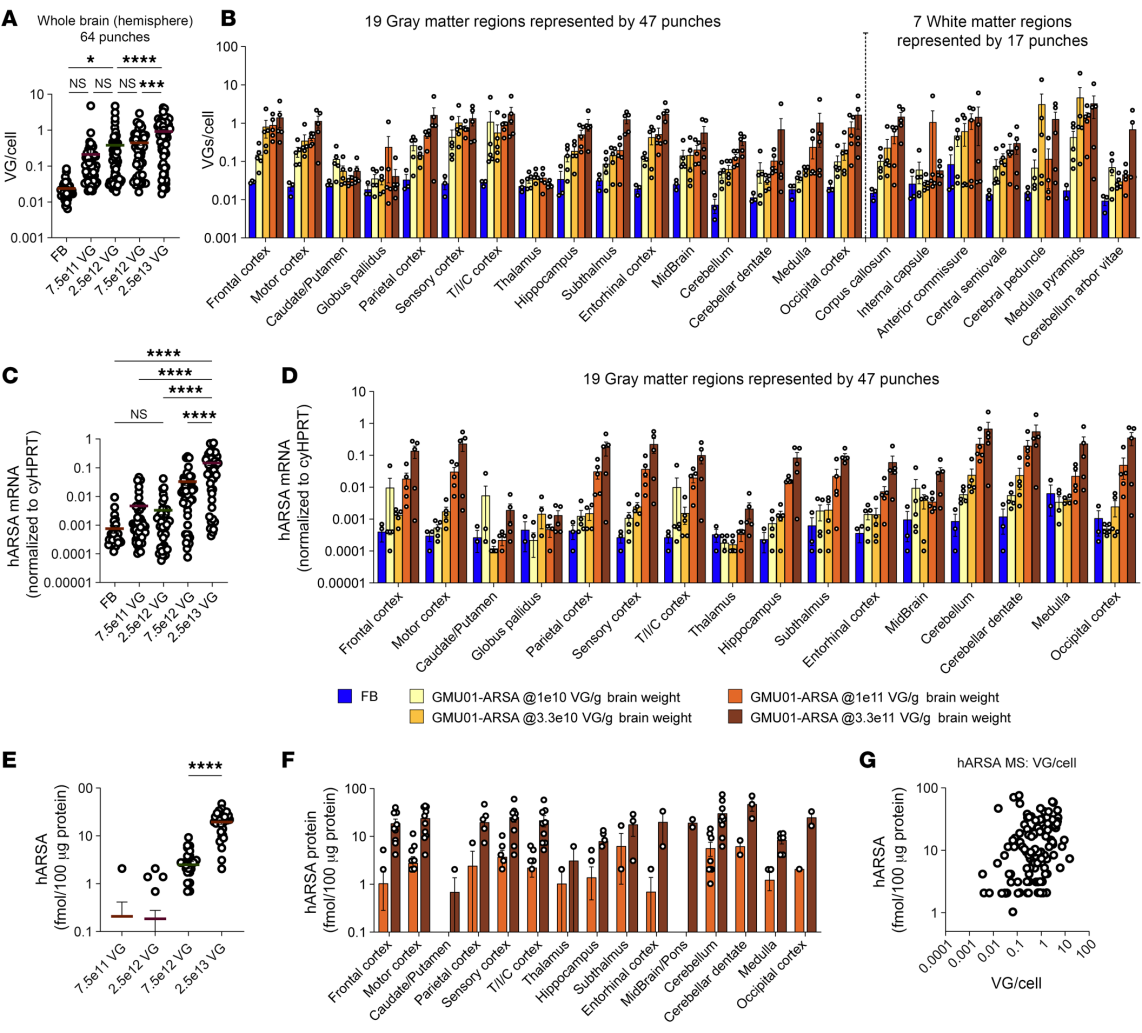
Widespread dose-dependent vector biodistribution and ARSA expression in NHP brain. Purpose-bred, naive, male/female cynomolgus (Cambodia 2 to 3 years old, 2.6 to 3.1 kg) NHPs seronegative for AAV.GMU01 neutralizing antibodies were dosed by single direct ICM infusion. Animals received a single 2.5 mL infusion of AAV.GMU01-*ARSA* at 0.125 mL/min, followed by a 250 μL flush with formulation buffer. Five weeks after dose, the animals were euthanized, and samples were assessed. (**A** and **B**) Vector biodistribution (digital PCR [dPCR]) normalized to the *TUBB1* gene intron. Each data point represents VG/cell exposure for that tissue punch, either (**A**) averaged across all NHPs in that group or (**B**) presented by individual brain regions. (**C** and **D**) *ARSA* mRNA (RT-dPCR) normalized to endogenous *HPRT* gene. Each data point represents normalized ARSA expression in that tissue punch, either (**C**) averaged across all NHPs in that group or (**D**) presented by individual brain regions. (**E** and **F**) Human ARSA protein expression (liquid chromatography–mass spectrometry). Each data point represents the amount of human ARSA in that tissue punch, averaged across 3 animals in that group, either presented as an (**E**) average across all NHPs in that group or (**F**) presented per individual brain region. Data below lower limit of quantitation have been excluded. (**G**) Correlation of ARSA protein and VG/cell at 7.5e12 and 2.5e13 VG/NHP doses (*R*^2^ = 0.06 and 0.18, respectively). Samples represent 64 biopsy punches from brain representing 19 distinct gray matter regions and 7 distinct white matter regions. Data are shown as the mean ± SEM. Two-way ANOVA with Tukey’s multiple-comparison test. *P < 0.05; **P < 0.01; ***P < 0.001; ****P < 0.0001. FB, formulation buffer; T/I/C cortex, temporal/insular/cingulate cortex.

To validate our therapeutic approach in a large-animal model, we examined the biodistribution and tolerability of AAV.GMU01-*ARSA* in a NHP model. AAV.GMU01-*ARSA* was delivered via ICM infusion to 2- to 3-year-old cynomolgus NHPs at 4 doses ranging from 7.5e11 to 2.5e13 VGs/animal (study design in Supplemental Table 6). Neurological and behavioral tests were performed before dose, 7 days after dose, and at necropsy (day 35). Tissue, CSF, and plasma were collected for analysis of VGs, *ARSA* mRNA, and ARSA protein. A dose-dependent increase in AAV.GMU01-*ARSA* vector biodistribution was observed throughout the brain (Figure 6A) and gray and white matter punches collected from 19 and 7 brain regions, respectively (Figure 6B). This correlated with dose-dependent increases in *ARSA* mRNA (Figure 6, C and D) and protein levels (Figure 6, E–G) at the 7.5e12 and 2.5e13 VG/animal doses. Additionally, uniform, dose-dependent vector biodistribution (Supplemental Figure 12A) and ARSA expression (Supplemental Figure 12B) were observed in DRGs and spinal cord along the rostral-caudal axis. We also noted a dose-dependent increase in vector biodistribution in liver, spleen, and cervical lymph nodes (Supplemental Figure 12C).

**Figure 7:**
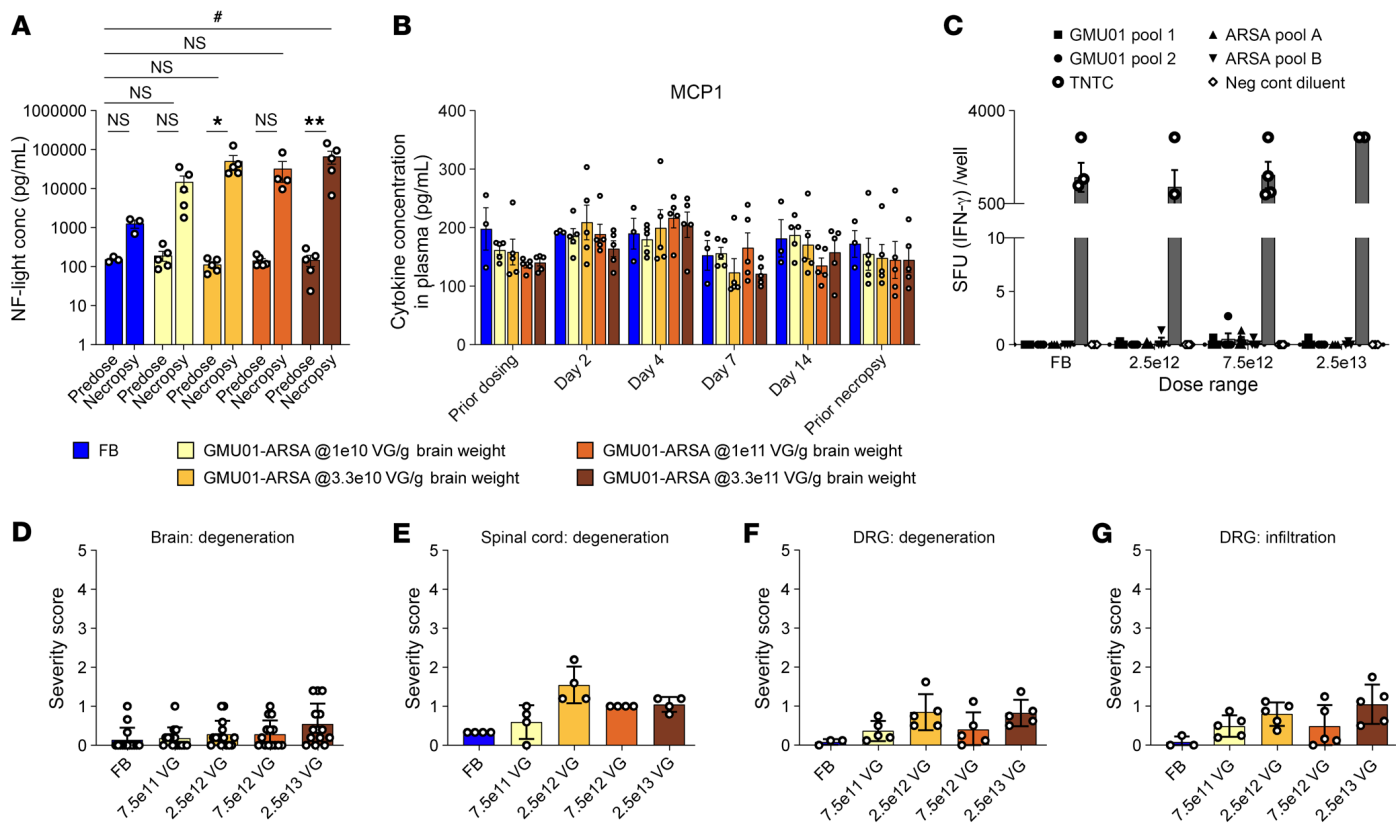
ICM infusion was well tolerated, resulted in expected Nf-L elevation, and did not trigger innate nor cell-mediated immune responses. (**A**) Predose and at necropsy, CSF was collected and analyzed for Nf-L levels by the Simoa platform (Quanterix). Each data point represents Nf-L levels per animal, averaged across all NHPs in that group. (**B**) Plasma was isolated predose at days 2, 4, 7, 14 after dose, and at necropsy. The Luminex assay was used to determine the concentration of MCP1 (graphed) and IL-1b, IL-1RA, IL-6, IL-10, IL12/23 (p40), IL-15, IL-18, IFN-γ, TNF-α, G-CSF, MCP-1, MIP-1b, GM-CSF, IL-2, IL-4, IL-5, IL-8, IL-13, and IL-17A (graphed in Supplemental Figure 13). Each data point represents cytokine concentration in that sample, averaged across all NHPs in that group. (**C**) PBMCs isolated from animals in the 1e11 and 3.3e11 VG/g brain weight dosing groups were subjected to IFN-γ enzyme-linked immunosorbent spot. (**D**–**F**) Histopathological findings in brain, spinal cord, and DRG; each data point represents the maximum severity of findings scored on 1–2 sections per animal. Severity scores refer to findings graded as 0 = no findings, 1 = minimal, 2 = mild, 3 = moderate, 4 = marked, and 5 = severe. Data are shown as the mean ± SEM; 2-way ANOVA with *Tukey’s and ^#^Dunnett’s multiple-comparison test.

Clinical signs (functional or behavioral deficits) were absent in NHPs at 1 or 5 weeks (necropsy) after dose in either dosing group (Supplemental Table 7). ICM infusion led to an increase in CSF Nf-L levels, which was more pronounced in the AAV.GMU01-*ARSA*–treated animals; the increase was not dose dependent (Figure 7A). No significant change in plasma cytokine concentrations was observed at any dose (Figure 7B and Supplemental Figure 13), and IFN-γ enzyme-linked immunosorbent spot indicated the absence of cell-mediated immune responses to either the GMU01 capsid or ARSA protein at all doses (Figure 7C). Subclinical microscopy findings were observed in the brain, DRG, spinal cord, and peripheral nerves (Figure 7, D–G, and Supplemental Figure 14), including scant degenerate neurons surrounded by gliosis present in the cortex, cerebellar Purkinje cells, and rarely in the thalamus (Figure 7D and Supplemental Figure 14A). Neuronal degeneration in the spinal cord consisted of nerve fiber degeneration, while in peripheral nerves, degeneration phenotype of the sciatic, femoral, and radial nerves was also observed (Figure 7E and Supplemental Figure 14, B and C). Neuronal degeneration in the DRG was noted at all test article doses (Figure 7F and Supplemental Figure 14, D and E). No test article–related findings were observed in visceral organs (heart, liver, gallbladder, spleen, pancreas, adrenal gland, lung, bone, sternum/marrow, ovary, duodenum, testis, epididymis, thymus, eye, uterus with cervix, and kidney) at any dose (Supplemental Figure 14F).

In conclusion, single administration of AAV.GMU01-*ARSA* by direct ICM injection results in widespread ARSA expression in CNS and is well tolerated in cynomolgus monkeys.

### AAV.GMU01-ARSA treatment results in potentially therapeutic levels of ARSA expression

**Figure 8:**
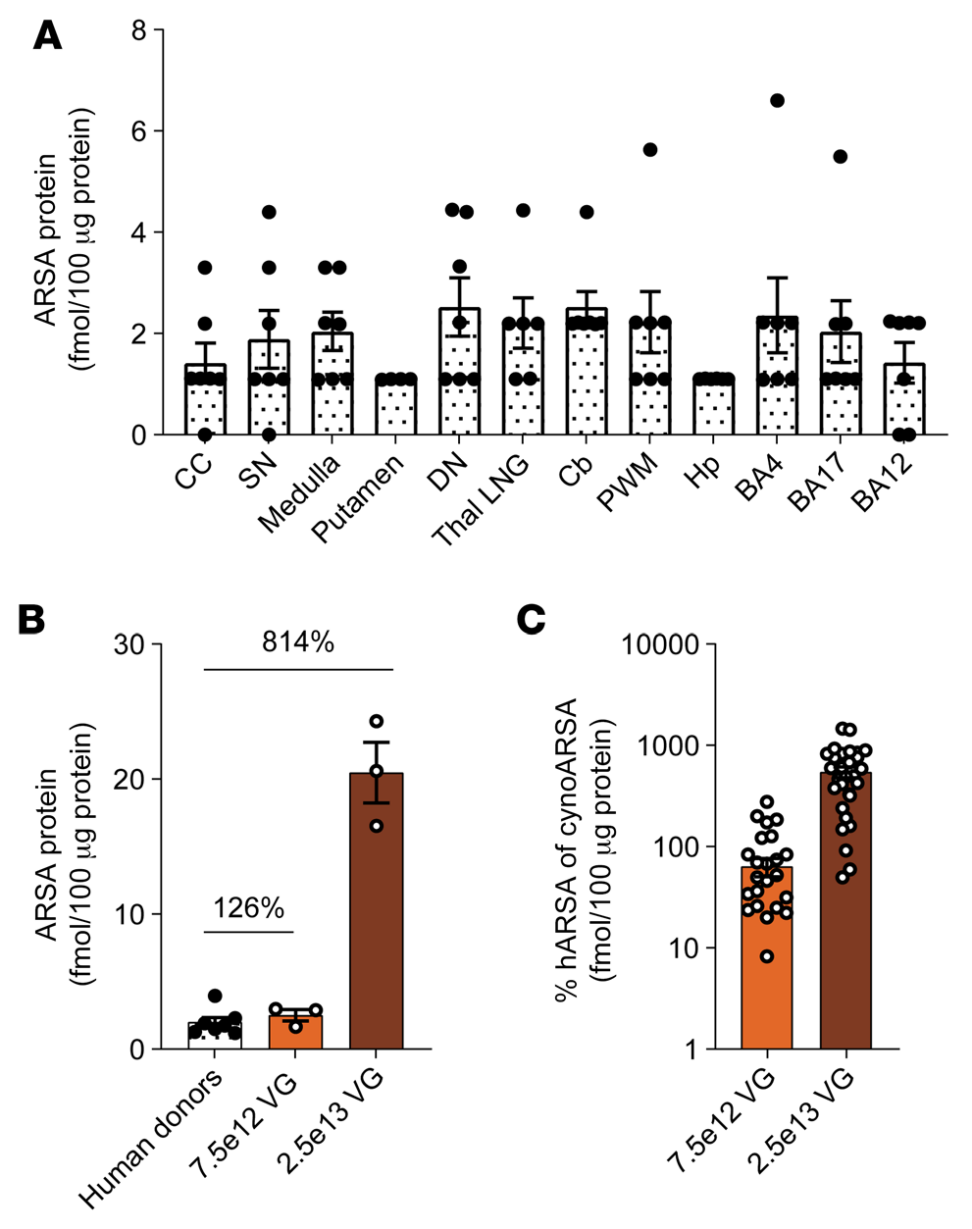
AAV.GMU01-*ARSA* effective doses are at potentially therapeutic levels. (**A**) Liquid chromatography–mass spectrometry (LC-MS) was performed to quantify human ARSA protein levels in 12 brain regions from 7 healthy 3- to 8-year-old organ donors. Human tissue was received from the NIH NeuroBioBank at the University of Miami and the Sepulveda Research Corporation. gm, gram. (**B**) LC-MS was performed to quantify human ARSA protein levels in 30 tissue biopsy punches collected from brain (gray matter) of NHPs in dose-ranging study. Mean ARSA protein in each group is presented. (**C**) LC-MS was performed to quantify both human and endogenous *Macaca fascicularis* cynoARSA protein levels. Each data point is represented as a ratio of human ARSA to cynomolgus cynoARSA protein in that tissue punch and averaged across 3 animals in that group. Data are shown as the mean ± SEM. CC, corpus callosum; SN, substantia nigra; DN, dentate nucleus; Thal LNG, lateral geniculate nucleus of the thalamus; Cb, cerebellum; PWM, periventricular white matter; Hp, hippocampus; BA, Brodmann area.

Diminished ARSA function without clinical symptoms, referred to as ARSA pseudodeficiency, is present in approximately 1% to 2% of the global population (55, 56). Genetic and phenotypic comparisons indicate that individuals with ARSA enzyme levels between 5% and 20% of normal are largely asymptomatic (14, 57–61). To investigate the potential for AAV.GMU01-*ARSA* treatment to achieve therapeutic ARSA levels, we measured postmortem ARSA protein in the brains (12 brain regions) of 7 healthy human donors aged between 3 and 8 years old. Human brain regions from the 7 age-matched donors showed an average of 1.9 fmol ARSA protein per 100 μg total protein (Figure 8A). In comparison, brain-wide mean human ARSA protein levels in NHPs were 2.5 fmol (7.5e12 VG/NHP) and 20.4 fmol (2.5e13 VG/NHP) per 100 μg total protein (Figure 6E). This corresponded to 1.2- and 8.1-fold higher ARSA protein levels than in the human samples (Figure 8B). Within-sample comparisons of human ARSA and endogenous cynomolgus ARSA (cynoARSA) protein levels across 19 brain regions indicated that human ARSA protein levels were approximately 63% and 546% higher than the endogenous cynoARSA (Figure 8C) at the 7.5e12 and 2.5e13 VG/NHP doses, respectively.

We conclude that AAV.GMU01-*ARSA* treatment in NHPs results in clinically promising ARSA protein expression in the CNS.

## Discussion

AAV vector gene therapy represents a promising approach for treating genetic diseases due to its ability to deliver sustained transgene expression, particularly within the CNS. Early evidence supporting the potential efficacy of ARSA replacement therapy in MLD emerged from studies in mouse models, where high doses of i.v. administered recombinant ARSA were shown to decrease sulfatide levels (62). However, enzyme replacement therapy (ERT) has demonstrated limited success in patients, primarily due to its inability to effectively cross the blood–brain barrier (63). Lentiviral hematopoietic stem cell gene therapy (HSC-GT) has shown considerable efficacy in early-stage MLD, offering a continuous source of ARSA production. However, this approach is associated with substantial risks stemming from the myeloablative conditioning required for transplantation (22). In contrast, AAV-mediated ARSA replacement therapy addresses these limitations by providing a safer, minimally invasive alternative. Preclinical studies across cellular, small-animal, and NHP models have demonstrated the efficacy and safety of AAV-mediated ARSA delivery (62, 64–68).

The ability of AAV vectors to facilitate stable and sustained ARSA expression from a single administration promises long-term therapeutic benefits without the need for repeated treatments. This approach eliminates the peak-to-trough fluctuations in enzyme activity observed with ERT while circumventing the procedural risks associated with HSC-GT. Consequently, AAV-mediated ARSA replacement offers a robust and practical strategy for addressing the neurological and systemic manifestations of MLD.

### Advantages of AAV.GMU01 for ARSA delivery.

AAV.rh10-mediated gene replacement alleviated many long-term MLD-associated phenotypes in a mouse model of MLD (33–35) but failed to demonstrate efficacy in the clinic despite long-lasting restoration of ARSA activity in the CSF (ClinicalTrials.gov NCT01801709) (69). This failure has been attributed to the inability of intracerebral delivery to achieve sufficient and widespread ARSA expression across the brain regions impacted by MLD (70). While well tolerated in NHPs, the practicality and tolerability of multiple intracerebral injections in patients with extensive white matter disease remain uncertain.

To overcome these limitations, AAV.GMU01 was specifically selected for its enhanced brain penetration, broad CNS biodistribution, and robust transgene expression in NHPs following a minimally invasive ICM delivery. This approach enables targeted expression of ARSA in affected brain regions, achieving near-normal levels of ARSA activity without the need for direct intracerebral administration, which may be poorly tolerated in pediatric MLD patients. While historically considered a higher-risk procedure, recent advances in image-guided techniques and catheter design have improved the safety and feasibility of ICM delivery, particularly in pediatric settings. The clinical feasibility of ICM delivery is further supported by its use in ongoing trials for similar CNS conditions, including phase 3 trials for GM1 gangliosidosis (ClinicalTrials.gov NCT04273269) and MPS type II (ClinicalTrials.gov NCT03566043) in young children. Early-phase trials in adults with CNS disorders such as frontotemporal dementia (ClinicalTrials.gov NCT04747431 and NCT04408625), Parkinson’s disease (ClinicalTrials.gov NCT04127578), MPS type I (ClinicalTrials.gov NCT03580083), and Gaucher type II (ClinicalTrials.gov NCT04411654) also demonstrate its broad applicability. Nevertheless, ICM delivery still requires specialized expertise and clinical infrastructure, and its procedural risks must be carefully considered in the clinical context. Ongoing clinical studies will be instrumental in further defining its safety profile across patient populations.

Importantly, intrathecal administration of AAV.GMU01 in NHPs resulted in lower vector exposure across the brain compared with AAV.rh10, while achieving superior biodistribution and transgene expression. This enhanced efficiency supports the potential for dose reduction, mitigating AAV-associated toxicity without compromising therapeutic efficacy. These attributes position AAV.GMU01 as a promising capsid for addressing the limitations of previous approaches and advancing ARSA gene replacement therapy for MLD.

### Preclinical validation in mouse and NHP models.

To evaluate the therapeutic potential of AAV.GMU01-*ARSA*, we conducted comprehensive pharmacology and efficacy studies in both mouse and NHP models. In mice, 4 studies were designed to model therapeutic intervention at various stages of neuropathological progression, reflecting presymptomatic and early-symptomatic MLD patients. Pharmacology studies in neonatal mice corresponded to the developmental age of the youngest intended patient population.

In the absence of ARSA activity, *Arsa*-KO mice exhibited progressive sulfatide accumulation in the CNS and visceral organs, gliosis, microglial activation, and histopathological abnormalities, including degeneration and vacuolation in brain parenchyma. Auditory dysfunction, a hallmark of MLD, was also present in these models. Treatment with AAV.GMU01-*ARSA* restored ARSA activity to WT levels in various tissues, normalizing sulfatide levels, reducing neuroinflammatory markers, and reversing hearing impairment. Longitudinal studies demonstrated consistent ARSA-mediated sulfatase activity and sustained sulfatide reduction in the brain, spinal cord, CSF, and plasma. These findings support the applicability of CSF and plasma biomarkers for monitoring therapeutic efficacy.

Dose-ranging studies revealed robust increases in sulfatase activity and reductions in sulfatide deposits in neonatal and early-neuronopathic mice, supporting the therapeutic potential of this approach and informing dose selection for NHP studies. Notably, cross-correction extended the therapeutic reach of ARSA, with over 84% of the brain showing evidence of corrected cells, despite fewer than 10% being directly transduced. This process, mediated by uptake of extracellular enzyme via the cation-independent mannose-6-phosphate receptor (50–52), underscores the broad applicability of AAV.GMU01-*ARSA*.

Although bulk tissue assessments do not provide cell-specific resolution, our data suggest that AAV.GMU01 facilitates more productive transduction events compared with other capsids. This is consistent with prior studies, such as those by Goertsen et al., in which AAV.CAP-B10 achieved higher expression per cell despite lower cell transduction than AAV-PHP.eB, and AAV.CAP-B22 outperformed AAV.CAP-B10 in brain expression despite similar DNA exposure (71). Kondratov et al. reported discrepancies between VG exposure and mRNA expression in a 29-capsid AAV library for the NHP CNS, showing that AAV.2i8 achieved higher expression than AAV5 at lower vector exposure (71), highlighting capsid efficiency as a key factor in improving therapeutic outcomes while mitigating AAV-associated toxicity.

In NHP studies, AAV.GMU01-*ARSA* was delivered via ICM infusion at varying doses. Dose-dependent increases in vector biodistribution and human ARSA mRNA and protein levels were observed throughout the CNS. Remarkably, human ARSA protein levels in NHP brains exceeded 126% of endogenous levels measured in healthy human donors at 1e11 VG/g brain weight and over 800% at 3.3e11 VG/g brain weight. Such levels surpass those associated with ARSA pseudodeficiency, where individuals remain asymptomatic despite significantly reduced enzyme activity (14, 55–60). These findings suggest that even modest restoration of ARSA activity could have profound therapeutic effects in MLD patients, enabling effective treatment at lower doses.

The therapeutic potential of AAV.GMU01-*ARSA* was reinforced by its tolerability in NHPs. Although neuronal degeneration and mononuclear cell infiltrates were observed in some tissues, they were not associated with functional or behavioral deficits. Increased CSF Nf-L levels were detected after treatment but were not dose dependent, and no significant changes in plasma cytokine levels or cell-mediated immune responses were observed. These results suggest a favorable safety profile for AAV.GMU01-ARSA.

### Challenges and limitations.

While these preclinical studies highlight the promise of AAV.GMU01-*ARSA*, several areas warrant further investigation. The lack of cell-specific resolution in transgene expression assessments underscores the need for future detailed studies on cellular tropism. Moreover, GLP toxicology studies using clinically representative materials are essential for advancing this therapy to human trials.

Gene therapy inherently results in a mosaic distribution, with only a subset of transduced cells producing WT or elevated levels of ARSA to compensate for nontransduced cells. This mosaicism highlights the challenge of replicating natural low-activity states, such as those seen in individuals with ARSA pseudodeficiency, through therapeutic interventions. This limitation likely contributes to the incomplete normalization of sulfatide metabolites observed in this study, despite restored ARSA activity. Additionally, differences in sulfatide metabolism dynamics between mice and humans may contribute to incomplete sulfatide clearance in preclinical models, even when sufficient enzyme activity is present.

Cross-correction, where ARSA is taken up by nontransduced cells, is a key mechanism of therapy but may vary in efficiency across cell types, necessitating broader transduction or higher enzyme activity to achieve complete correction. Moreover, disease kinetics and specific metabolic demands in mouse models may not fully replicate human pathology. For example, murine ARSA exhibits approximately 3.5 times higher activity than human ARSA (72), potentially influencing the therapeutic response. Understanding the interplay between enzyme distribution, activity thresholds, and tissue-specific requirements will be essential for optimizing future therapeutic strategies.

Nf-L serves as a sensitive biomarker for neuronal injury, neurodegeneration, and therapeutic efficacy in both preclinical and clinical settings. However, its interpretation in the context of AAV-based therapies is complex. Nf-L levels reflect neuronal damage or repair, which may lag behind metabolic changes, making it a delayed biomarker of disease progression or treatment response. In this study, Nf-L levels increased in both mouse and NHP models following AAV delivery, a phenomenon well documented in the field. It is important to note that AAV administration is often associated with transient Nf-L elevations, confounding its use as a biomarker of disease repair during the early posttreatment phase. For example, in NHPs treated with AVB-101 (AviadoBio), Nf-L levels increased after intrathalamic AAV delivery but returned to baseline in serum and approached baseline in CSF after 6 months. Similarly, in clinical trials of AMT-130 (uniQure), patients experienced Nf-L elevations after treatment that returned to near-baseline levels within 12–24 months, indicating a transient neuronal response to therapy. These observations highlight the need to interpret early Nf-L changes cautiously and to focus on long-term trends to assess therapeutic efficacy and safety accurately.

### Immune considerations.

Human ARSA shares 96% similarity with cynomolgus monkey ARSA, raising the possibility of cross-reactive immune responses even in WT animals. While this high level of similarity suggests that central tolerance could mitigate immunogenicity, it does not entirely eliminate the risk of an immune response to the human ARSA transgene. This highlights the complexity of interpreting immune responses across species and underscores the need for immune monitoring during preclinical and clinical development.

In addition, the cross-reactive immunologic material (CRIM) status is a critical factor in shaping treatment strategies for MLD. CRIM-negative individuals, who lack endogenous ARSA protein expression, may be at higher risk of developing an immune response to the ARSA transgene. However, comprehensive data on CRIM status in MLD patients are limited. This gap stems partly from the challenges of measuring ARSA protein levels and the reliance on standardized dried blood spot sulfatide and ARSA enzyme assays in newborn screening, which primarily aim to identify individuals at high risk of developing MLD rather than evaluate CRIM status. Existing data suggest that CRIM-negative patients may represent a small subset of the MLD population. A newborn screening study in Minnesota analyzing 100,000 blood spots identified 73 screen-positive samples, including 51 with pseudodeficiency variants (reduced but detectable ARSA protein), 20 heterozygous for pathogenic or unknown variants (reduced but detectable ARSA protein), and 2 homozygous for potentially pathogenic mutations (likely CRIM negative) (73). This suggests that approximately 2.73% of newborns at high risk of MLD lack ARSA protein. Similarly, among European MLD patients, 3 common ARSA mutations were identified, with only 1 allele (allele I) associated with immunologically undetectable ARSA. Approximately 6% of MLD patients are homozygous for this allele, suggesting that 94% of MLD patients retain immunologically detectable ARSA (74, 75). These findings indicate that CRIM-negative individuals likely represent a small proportion of the overall MLD population, which has implications for clinical adoption of gene replacement strategies like the one described in this study.

Finally, while NHP studies provide critical insights into biodistribution and transgene expression, they have limitations in predicting human immune responses. Clinical experience has revealed risks such as complement activation and thrombotic microangiopathy (76, 77). Additionally, T cell–mediated responses against AAV capsid or transgene-expressing cells have been observed in both clinical and preclinical settings, contributing to DRG pathology and sensory neuron degeneration (26, 78). As such, careful clinical monitoring and consideration of immunomodulatory strategies will be important as this approach advances toward human trials.

### Summary.

This study highlights the therapeutic potential of AAV.GMU01-*ARSA* gene replacement therapy for MLD. Preclinical findings demonstrate that AAV.GMU01-*ARSA* effectively addresses key MLD-associated phenotypes, including sulfatide level normalization, reduction of neuroinflammation, and restoration of functional deficits, while maintaining a favorable safety profile in both mouse and NHP models. Notably, a single administration of AAV.GMU01-*ARSA* via ICM delivery in NHPs was well tolerated, achieving human ARSA expression at potentially therapeutic levels. These results provide a robust foundation for progressing to large-animal toxicology assessments with clinically representative materials. However, translating these promising preclinical outcomes to clinical applications requires careful consideration. Variations in enzymatic activity, biodistribution, and disease progression between species highlight the need for rigorously designed clinical trials to validate the safety and efficacy of this therapy in patients. In conclusion, AAV.GMU01-ARSA offers considerable promise as a long-term, minimally invasive treatment for MLD, with the potential to transform patient outcomes and improve the lives of affected individuals and their families.

## Methods

### Sex as a biological variable

Both male and female animals were included in all in vivo studies unless otherwise stated. Experimental groups were balanced by sex when feasible, and data were analyzed to assess potential sex-based differences. No significant differences attributable to sex were observed in the key outcome measures; therefore, data from both sexes were pooled for analysis and presentation.

### Vector design

Three AAV constructs were used: (a) CBA-ARSA-WPRE-bGH, expressing human ARSA under a ubiquitous CBA promoter with WPRE and bGH poly(A) (4,397 nt); (b) CBA-nLuc-Flag-T2A-NLS-mCherry-bGH, coexpressing FLAG-tagged nLuc and mCherry (4,537 nt); and (c) CBA-eGFP-bGH, expressing eGFP (4,380 nt). All vectors were produced by transient transfection in HEK293 cells.

### Animal models and AAV administration

#### Rodent studies.

The model is listed (The Jackson Laboratory) as B6N.129P2(CBA)-Arsatm1Gie/J (ARSA^–/–^). Disruption of the Arsa gene results in the progressive accumulation of sulfatide species in visceral organs and the CNS, similar to the Gieselmann model (12, 77, 79). Gliosis and microglial activation were observed, with a notable auditory phenotype in line with the loss of neurons of the ventral cochlear nucleus, posterior part, and spiral ganglion, as seen in the Gieselmann model (12). Mice received ICV injections of AAV.GMU01-ARSA or formulation buffer at varying ages (neonatal and 2, 6, and 13 months) and doses (up to 3.3e11 VG/g brain). All animals used in this manuscript were bred and housed, and experiments performed at The Jackson Laboratory.

#### NHP studies.

Cynomolgus monkeys (2–3 years old, 2–3 kg) received AAV via lumbar intrathecal catheter, ICV, or ICM injections. In dosing studies, animals received 2.5 mL of vector (0.125 mL/min) with formulation buffer flush.

### Human tissue

Human tissue was acquired from the NIH NeuroBioBank at the University of Miami and the Sepulveda Research Corporation.

### Histopathology and IHC

Formalin-fixed NHP tissues were embedded in paraffin embedded and stained with H&E for pathology review. GFP immunohistochemistry was conducted on FFPE slides with antigen retrieval and DAB detection.

### Biochemical and molecular assays

Tissue homogenates were used for sulfatase activity (p-Nitrocatechol sulfate hydrolysis), nanoluciferase activity (Nano-Glo), total protein (bicinchoninic acid), and lipid extraction for sulfatide liquid chromatography–mass spectrometry (LC-MS). VG copies were quantified by digital PCR (dPCR). RNA was extracted and analyzed by reverse transcription-dPCR for ARSA, inflammatory markers, and lysosomal genes.

### Proteomics and imaging

LC-MS–quantified ARSA protein in rodent and NHP tissues using high-field asymmetric waveform ion mobility spectrometry parallel reaction monitoring with isotope-labeled peptides. FISH and IHC codetection assays were used to visualize WPRE RNA and transgene proteins alongside cell-type markers.

### Statistics

Various statistical tests were used in this work to analyze the data, as noted in the figure legends. We widely applied 1- and 2-way ANOVA, either with Tukey’s, Šidák’s, or Dunnett’s test, where appropriate. Correlation analysis was applied to assess the relationship between VG exposure and transgene expression or protein levels. All data in graphs are shown as the mean ± SEM.

### Study approval

All experiments were conducted in Association for Assessment and Accreditation of Laboratory Animal Care International accredited institutions. Animals were handled in accordance with the rules and regulations of the IACUC, in compliance with the Animal Welfare Act, and adhered to principles stated in the Guide for the Care and Use of Laboratory Animals. All efforts were made to minimize pain and distress in these purpose-bred animals. Only purpose-bred naive cynomolgus NHPs were used in studies. NHPs were prescreened for AAV neutralizing antibodies, and seronegative animals were selected for the studies.

### Data availability

All data used to generate graphs are included in the Supporting Data Values file that accompanies this manuscript. The single-nucleus RNA sequencing data supporting the findings of this study are openly available in the Gene Expression Omnibus database under accession number GSE299162. Details of all assays and procedures are provided in the Supplemental Methods.

## Author contributions

SR, MG, and CM conceived the study. SR, JA, JB, LG, DG, YC, SA, QT, CO, BZ, and GG designed the methodology. JA, JB, MR, LG, DG, MH, YC, EW, SA, QT, RT, YL, JMH, DMD, EC, JGFA, AMR, SN, BN, DW, SCC, and MT performed the investigations. JA, JB, DG, SA, QT, JMH, JGFA, MT, and SCC curated the data. SR, JA, DG, SA, JMH, JGFA, BE, MT, GG, and SCC analyzed the data. SR, JA, SA, JMH, JGFA, BE, MT, and SCC contributed to data visualization. SR, MG, and CM supervised the study. SR, JA, and JB wrote the original draft. SR, MG, BE, and JMH reviewed and edited the manuscript.

## Supplementary Material

Supplemental data

Supporting data values

## Version 1 06/19/2025

In-Press Preview

## Version 2 08/15/2025

Electronic publication
