# Supplementary material for: Cross-species efficacy of AAV-mediated ARSA replacement for metachromatic leukodystrophy
Source: J Clin Invest. 2025 Jun 19;135(16):e185001. doi: 10.1172/JCI185001 (PMC12352910; doi:10.1172/JCI185001)
Supplement: Supplemental data [file jci-135-185001-s187.pdf]

SUPPLEMENTARY METHODS

Vector design:

CBA-ARSA-WPRE-bGH: A 4397 nucleotide payload comprised of AAV2 Inverted Terminal Repeats (ITRs), a 1.7kb ubiquitous CBA promoter (CMV enhancer, chicken β-actin promoter, and a chicken β-actin/rabbit β-globin hybrid intron) expressing a functional copy of the human ARSA gene (ARSA), and having the bGH polyA and termination signal. The transgene also incorporates an engineered Woodchuck Hepatitis Virus Posttranscriptional Regulatory Element (WPRE) to enhance ARSA expression in transduced cells.

CBA-Nanoluciferase-Flag-T2A-NLS-mCherry-bGH: A 4537 nucleotide payload comprised of AAV2 ITRs, a 1.7kb CBA promoter expressing both a FLAG-tagged Nanoluciferase (Nluc) and mCherry separated by the T2A ribosomal skipping peptide from a common ORF sequence with the bGH polyA and termination signal.

CBA-eGFP-bGH: A 4380 nucleotide payload comprised of AAV2 ITRs, the CBA promoter expressing a eGFP ORF and having the bGH polyA and termination signal.

In vivo studies were performed utilizing material manufactured by transient transfection in adherent HEK293 cells.

ARSA knockout mouse model: The model used mirrors the Gieselmann model (48) and was commissioned by Sanofi through Regeneron in 2012. The model is listed (Jackson Labs) as B6N.129P2(CBA)-$^{\text{Arsatm1Gie/}}$J (*ARSA*$^{-/-}$) and screened by "Loss of Native Allele (LOA) assay (77). The model was created through a disruption of *Arsa* Exon 4 near HindIII site (Genomic coordinates NCBIM37: chr15:89,302,907-89,307,855) with a loxP-hUBp-em7-Neo-polyA-loxP cassette in C57BL6 embryonic stem cell line, resulting in a positive clone (B-E9) with a Deletion of 44 bp for genotyping purposes NCBIM37:chr15:89,305,085-89,305,128. Disruption of the *Arsa* gene results in the progressive accumulation of sulfatide species in visceral organs, and the central nervous system, similar to the Gieselmann model (12, 78, 79). Gliosis and microglial activation is observed, with a notable auditory phenotype in line with the loss of neurons of the

ventral cochlear nucleus, posterior part, and spiral ganglion, as seen in the Gieselmann model (12, 80). Rodent study designs included in **Supplementary Tables 3-5**.

NHP Studies: For ICV injections, animals were anesthetized and placed in the integrated head fixation frame with the ClearPoint (ClearPoint Neuro) array base placed on the cranium. The targeted dosing regions and cannula trajectory were determined using MRI scans. Prior to surgery, approximately 1 mL of CSF was removed. Bilateral ICV injections were perfumed sequentially under MRI guidance using SmartFlow NGS-NC-05 cannula (ClearPoint Neuro). A total of 1 mL of AAV.GMU01-NLuc-mCherry was administered at 0.125 mL/min flow rate into each lateral ventricle. For direct cisterna magna (ICM) injection, animals were placed in Trendelenburg position throughout dosing and for up to 15 minutes following dosing completion. Prior to dosing, approximately 1 mL of CSF was removed. A 24-gauge needle was inserted into the cisterna magna under fluoroscopy guidance. Animals were infused with a total of 2.0 mL AAV.GMU01-NLuc-mCherry at 0.125 ml/min flow rate while under anesthesia; A flush volume of 0.250 mL formulation buffer was given at the end of the dosing and the needle was left in place for at least 1-3 minutes before removal. Following the procedure, veterinary care was given for recovery. NHP study designs included in **Supplementary Tables 1-2 and 6.**

NHP sample collection: At necropsy, animals were perfused with chilled PBS pH 7.4, and their brains were cut in a brain matrix into 4 mm coronal slices and hemisected. The left hemispheres were frozen on dry ice for biochemical analysis, while the right hemispheres were fixed in 10% neutral buffered formalin for 36-48 hours at room temperature before embedding in paraffin blocks for histopathology and immunohistochemistry analysis. The spinal cord and DRGs from 4 levels (cervical, upper thoracic, lower thoracic and lumbar), as well as peripheral tissues were collected as frozen and fixed samples. Tissue punches were collected using 3 mm biopsy punches from indicated brain regions on the frozen brain slices, as well as from 4 levels of spinal cords, DRGs and peripheral tissues. Paraffin tissue sections were stained for hematoxylin and eosin and submitted for histopathology analysis.

NHP Histopathology: Pathology evaluations were conducted by a board-certified veterinary pathologist at HistoWiz (Long Island City, NY) or StageBio (Mt Jackson, VA) on hematoxylin and eosin-stained slides. For each animal, eight key brain regions (cerebral cortex, basal ganglia,

thalamus, hippocampus, midbrain/pons, cerebellum, medulla and major white matter tracts), as well as spinal cord and DRGs from cervical, upper thoracic, lower thoracic and lumbar levels were examined for axon/neuron degeneration and/or inflammation/cell infiltration. A minimum of 8 DRGs (2 from each level) were evaluated. Microscopic findings were graded as 0 for absence of lesion, 1 for minimal, 2 for mild, 3 for moderate, 4 for marked, and 5 for severe.

NHP GFP Immunohistochemistry (IHC): For GFP IHC, antigen retrieval was performed on FFPE slides from brain using EDTA solution (pH.9.0) for 20 minutes at 90°C. After blocking with 3% hydrogen peroxide for 10 minutes and 5% horse serum for 45 minutes, the slides were incubated with GFP antibody (ThermoFisher A-11122) at 1:500 dilution for one hour at room temperature, followed by incubation with anti-rabbit HRP (Abcam ab6721) at 1:200 for one hour at room temperature. The color development for GFP signal was achieved by incubating slides in DAB solution (Thermofisher 34002) for 3 minutes at room temperature. Brightfield images were captured by an Aperio AT2 image scanner.

***Tissue homogenization*:** Tissues were homogenized at 4C in cold TE buffer (10mM Tris pH7.4, 1mM EDTA) in 2mL tubes containing 1.4 mm ceramic beads using an Omni Beadruptor set for 20 second cycles 4.7 oscillation/sec. Following homogenization, aliquots were frozen at –80C until use.

***Tissue solubilization for Sulfatase Activity*:** To homogenized tissue, Nonidet P-40 was added to 0.1% final concentration, allowed to solubilize at 4C for 1.5 hours on an orbital shaker, then centrifuged at 18,000 *x g* for 20 minutes. Supernatant was removed and transferred to fresh Eppendorf tube on ice prior to BCA and Sulfatase activity assays.

***BCA Assay*:** Total protein concentration determined by BCA (bicinchoninic acid) assay (Thermo Scientific 23227) using 10ul supernatant diluted in water. Colorimetric detection of the cuprous cation (Cu^{1+}) by bicinchoninic acid (BCA) by absorbance at 562 nm. Molecular Devices SpectraMax 340PC-384 with SoftMax Pro version 5.4.4 software used to read 96 well microtiter plate.

***Nanoluciferase activity**:* Crude tissue homogenates were assayed for nanoluciferase activity using the Nano-glo luciferase assay system (Promega) according to manufacturer's instructions.

Luminescence was read using the Cytation C10 and was normalized to total protein content as measured using the BCA assay.

***Sulfatase Activity*:** Ten microliters of clarified supernatant was assayed for total sulfatase activity using sulfatase activity assay for hydrolyzed 4-Nitrocatechol (PNC) from 4-Nitrocatechol Sulfate (PNCS) substrate. (Abcam Ab204731). Activity determined by hydrolyzed 4-Nitrocatechol (PNC) of sample relative to PNC standard curve and read absorbance at 515 nm. Sulfatase activity reported as mU/mg (nmol/min/mg). Molecular Devices SpectraMax 340PC-384 with SoftMax Pro version 5.4.4 software used to read 96 well microtiter plate.

***Lipid extraction*:** An aliquot of tissue homogenate (or fluid) was extracted with 20-100X extraction solution (5 mM Ammonium Formate, 0.2% Formic Acid in Acetonitrile:Methanol (70:30), supplemented with 10 ng/ml C17-sulfatide). After mixing vigorously for 10min, samples were sit for 5min and vortexed again quickly (30s). All samples were centrifuged at 8,400 rpm for 10 min at 4°C. An aliquot of the supernatant (200uL) was transferred into a deactivated Q-sert Vial for LC-MS analysis. Standard curve (linearity range 0.03-1000 ng/mL, 2x- serial dilutions) were prepared in the same extraction solution using sulfatides with C16, C18, C24 and C24:1chain length (Matreya) and C18 2R-OH sulfatide and lyso-sulfatide (Avanti).

***LC-MS analysis of Sulfatides*:** A Waters Acquity UPLC system (Milford, MA) was coupled to a Qtrap 6500 mass spectrometer system (Framingham, MA) equipped with a ESI source operated in negative ion mode with the following parameters: curtain gas 25.0; ionSpray voltage -4.5 kV; temperature 500°C; ion source gas: 50 and 70; declustering potential -80V; entrance potential -10V; collision energy -155V; and collision cell exit potential -15V. The sulfatide species were separated on a Waters Acquity UPLC BEH Amide (1.7 μ, 2.1 x 100 mm, P/N: 186004801). The autosampler and column oven were maintained at 10°C and 20°C, respectively. The mobile phase consisted of Solvent A: 5 mM Ammonium Formate in 95:5 of Acetonitrile:Water, and Solvent B: 5 mM Ammonium Formate in 90:10 Methanol:Water. The gradient program started at 0% B and hold for 2min, followed a linear curve from 0%B to 100% B. After 1 more min at 100% B, it re-equilibrated at 0%B. All sulfatide species are normalized to the internal standard of C17-sulfatide and calculated by the standard curve of the corresponding standard sulfatides.

***GFP ELISA*: Measurement of GFP protein was completed using the GFP SimpleStep ELISA® Kit from Abcam (ab171581). Samples homogenized in TE buffer as described above were diluted in complete cell extraction buffer as follows: grey matter 1:5, spinal cord 1:5, heart: 1:5, liver 1:20. Samples were read at 450nm on a SpectraMax plate reader (Molecular Devices) using Softmax software.

***Genomic DNA (gDNA) isolation*: DNA was isolated from 50ul tissue homogenate using QIAmp 96 DNA QIAcube HT kit (cat# 51331) on a Qiacube HT according to manufacturer's protocol "QIAamp® 96 DNA QIAcube® HT Handbook". DNA concentration then was measured via absorbance with NANODROP 8000 (Thermo Fisher Scientific).

***Digital PCR*: Vector genome copies were determined from extracted DNA by dPCR using the QIAcuity Eight digital PCR System (Qiagen, Inc.) gDNA was analyzed in a 12ul reaction volume. Probes for bGH (AAV) and an endogenous mouse or cynomologous reference gene (Integrated DNA Technologies, Inc.) were used to measure gene copy number and calculate vector genomes per cell (2X bGH copies divided by reference gene copies.

***Total RNA isolation*: An aliquot of TE tissue homogenate was used for RNA extraction from the QIAcube HT (Qiagen, Inc.) using the RNeasy 96 QIAcube HT kit (#74171) according to manufacturer's protocol "RNeasy 96 Qiacube HT Handbook" with on-plate DNase digestion, with noted QIAzol (Qiagen #79306) chloroform RNA extraction according to the QIAgen "QIAzol Handbook" steps 1-7 preceding and transferring the supernatant to the S-block for QIAcube HT RNA purification.

RT-dPCR : Isolated total RNA copies were reverse transcribed to cDNA (Qiagen One Step Advanced Probe kit, Qiagen #250132) and quantitated for copies/ul using the QIAcuity Eight digital PCR System (Qiagen, Inc.) utilizing specific qPCR primer and probe sets for *hARSA* (Integrated DNA Technologies, Inc.), gene normalizer (*Hprt*, mouse or cynomologous specific), inflammatory markers (*Gfap, Aif1*), and marker of lysosomal health (*Lamp1*). Total reaction volume is 12ul per well. The 8.5k 96 well nanoplate (Qiagen #250021) was placed into QIAcuity instrument and RT-dPCR performed using manufacture suggested cycling. RNA copies for each

target gene were analyzed automatically by the software. Target gene was then normalized to the housekeeping gene using the QIAcuity software suite.

Cross correction analysis**:** Adjacent 5 μm FFPE sections were processed for either ARSA IHC and DAPI or *ARSA* ISH (against WPRE mRNA) and DAPI. Individual tiles were then stitched to obtain sagittal sections for both IHC and ISH images. These images were then registered globally with a transformation matrix (rotational and translational parameters) to align the IHC and ISH images. Individual tiles within the sagittal sections were then registered locally using DAPI to ensure good alignment between individual IHC and ISH tiles. In each tile, IHC positive cells and ISH positive cells were estimated by thresholding parameters empirically determined across the entire sagittal sections. In the case ISH, IHC positive cells had to have at least 20% overlap with a nuclei to be counted as a bonafide ISH signal. For IHC images, negative controls with no ARSA staining were used to determine thresholding parameters. Cross-correction factor which is the ratio of IHC positive cells to ISH positive cells was estimated for each tile and represented as a heat map.

ARSA LC-MS: Tissue homogenates from humans and non-human primates (NHPs) were processed for proteomic analyses using a stepwise protocol involving reduction, alkylation, and overnight digestion with Trypsin/Lys C. Sample handling was performed with the MINI 96 (Integra Biosciences) digital pipettor to enable high-throughput analysis. Digested peptides, spiked with synthetic heavy peptides labeled at the C-terminal R/K (13C15N), were clarified using EvoTip trap columns. These were connected online to an Evo 8 cm analytical column and separated using a preset 21-minute analytical gradient (60SPD) on the Evosep One nanoLC system. Absolute quantification of human and NHP ARSA proteins was attempted through an in-house multiplex LC-MS assay, leveraging high-field asymmetric waveform ion mobility spectrometry (FAIMS)-based gas-phase ion separation in high-resolution mode. Target peptide elution was empirically determined via chromatographic retention time (RT), and FAIMS-separated gas-phase ions were analyzed through parallel reaction monitoring (PRM).

For FAIMS-PRM, compensation voltages (CVs) for unique human and NHP peptides were optimized by ramping DC voltages from -50 CV to -30 CV to identify the optimal values for each peptide. While human brain homogenates followed a similar process, FAIMS was not

utilized for their analysis. MS1 data acquisition for all samples occurred at high resolution (240K), with tMS2 data acquired at 30K resolution. The accuracy and precision of the FAIMS-PRM assays were evaluated using a 12-point calibration curve with linear ranges of 0.02–50 fmol/µL for the human-unique peptide (QSLFFYPSYPDEVR) and 0.1–25 fmol/µL for the NHP-unique peptide (GGLPLEEVTLAEVLAAR). Quality controls (QCs) at low, medium, and high levels were prepared by spiking surrogate peptides into a pooled matrix. The ratio of endogenous to surrogate peptides was obtained from Skyline, with ARSA expression values calculated using a single-point calibration method:

$$C_{ARSA}\ (fmol/\mu L\ or\ nM) = [(1) \times T_{Vd}] \div B_{Vd}$$

R= L/H; L= endogenous and H= surrogate (heavy)

S_O= spike (H) on-column (fmol)

Vd0= digests volume (on-column)

T_{Vd} (µL) = total volume (µL) of starting materials

BVd (µL) = Total volume (µL) of brain homogenate (undigested) provided

To ensure precision and reproducibility, we achieved sub-nM quantification using a cutting-edge mass spectrometry platform, the Thermo Exploris 480 integrated with FAIMS and the Evosep nanoLC system. Isotope-labeled and unlabeled peptides for human-specific, NHP-specific, and shared peptide sequences were synthesized for calibration and QC. The human-unique peptide (QSLFFYPSYPDEVR) demonstrated a linear range of 0.02–50 fmol/µL, with a limit of quantification (LOQ) of 0.02 fmol/µL, while the NHP-unique peptide (GGLPLEEVTLAEVLAAR) exhibited a linear range of 0.1–25 fmol/µL and an LOQ of 0.1 fmol/µL. QC thresholds for high, medium, and low levels were defined as 5, 2.5, and 0.25 fmol/µL, respectively.

Sensitivity was validated through linear dynamic range and LOQ determination, with QC data consistently maintaining standard deviations within 10% for the human peptide and 17% for the NHP peptide, adhering to the FDA acceptability criteria of ±20%. These results highlight the assay's reliability, enabling accurate quantification of peptides at sub-nM concentrations and reinforcing its clinical relevance.

ELISpot procedure

- ***PBMC culture and immune stimulus:*** Frozen PBMCs were thawed at 37°C for 10 minutes and diluted in RMI-1640 medium containing 10% heat inactivated-FBS. Cells were plated at 100ul/well, in triplicate, to precoated 96 well ELISPOT plates purchased with anti-hIFN-gamma Single-Color ELISPOT kit (ImmunoSpot) for human (will cross react with NHP).
- ***Peptide Library and Control Preparations:*** Peptide libaries for the AAV.GMU01 capsid, and the hARSA gene product were generated by Mimetope and consisted of 15mer peptides spaced every 3 amino acids of the sequence. Each individual peptide was prepared in 80% DMSO at 50mg/ml.
- ***Positive Control Preparations:*** Ionomycin calcium salt from Streptomyces conglobatus [Sigma Aldrich I3909] and PMA [Phorbol-12-myristate-13-acetate – Calbiochem #5005820001] was used as positive controls. Stocks were prepared in DMSO and diluted in RMI-1640 medium to 4uM Ionomycin/100nM PMA
- ***ImmunoSpot hIFN g:*** Single-Color ELISPOT kit protocol was followed for staining and detection, as described by manufacturer. Plates were then imaged and counted on the C.T.L ImmunoSpot S6 Universal M2 analyzer with ImmunoSpot 7.0.28.4 Analyzer Proffesional DC; Immunospot 7.

Cytokine panel: Quantification of pro-inflammatory cytokines (IL-1β, IL-1RA, IL-6, IL-10, IL-12/23 (p40), IL-15, IL-18, IFN-γ, TNFα, G-CSF, MCP-1, MIP-1β, GM-CSF, IL-2, IL-4, IL-5, IL-8, IL-13 and IL-17A) in plasma was performed by Charles River using the standardized Luminex Assay.

NfL assessment: NF-L protein level was measured using Simoa NF-Light*TM* V2 Advantage kit from Quanterix (Item# 104073). Mouse plasmas sample were diluted 20-fold in kit sample diluent. NHP CSF sample were diluted 160-fold in Lysate diluent C reagent purchased from Quanterix (item# 103360). Diluted samples were analyzed on Simao HD-X analyzer.

Immunofluorescent RNA/Protein integrated co-detection assay: Co-detection of WPRE RNA with payload proteins (human arylsulfatase A or mCherry) and cell type markers from rodent and non-human primate (NHP) brain FFPE slides was performed using a Leica BOND RX

automated stainer (Leica Biosystems) . The multiplexing protocol was programmed to streamline *in situ* analysis of transgene RNA and a continuous IHC staining process for the co-detection of RNA and protein markers. The RNAscope 2.5 LS multiplex fluorescent kit (ACD, 322800) and the Bond polymer refine detection kit (Leica Biosystems, DS9800) were utilized in this automated protocol. Specific WPRE probes (rodent: ACD, 450268; NHP: ACD, 410058) targeted the 3' UTR of the transgene RNA during the *in situ* hybridization (ISH) step. Detection and visualization were achieved using TSA Vivid Fluorophore 647 (Biotechne, 75271KIT). Sequential IHC steps involved the use of antibodies against human ARSA (1:250, R&D Systems, AF2485) or mCherry (1:250, MAB131873), along with cell marker antibodies (1:500) for identifying neurons, astrocytes, microglia, and oligodendrocytes. DAPI was used for nuclear counterstaining. This integrated protocol enables precise co-detection of transgene RNA and protein with cell-specific markers, providing a comprehensive analysis of expression and cellular localization across rodent and NHP brain tissue samples. A full list of reagents can be found in **Supplementary Table 8.** Antibodies are listed in **Supplementary Table 9**.

SUPPLEMENTARY FIGURES

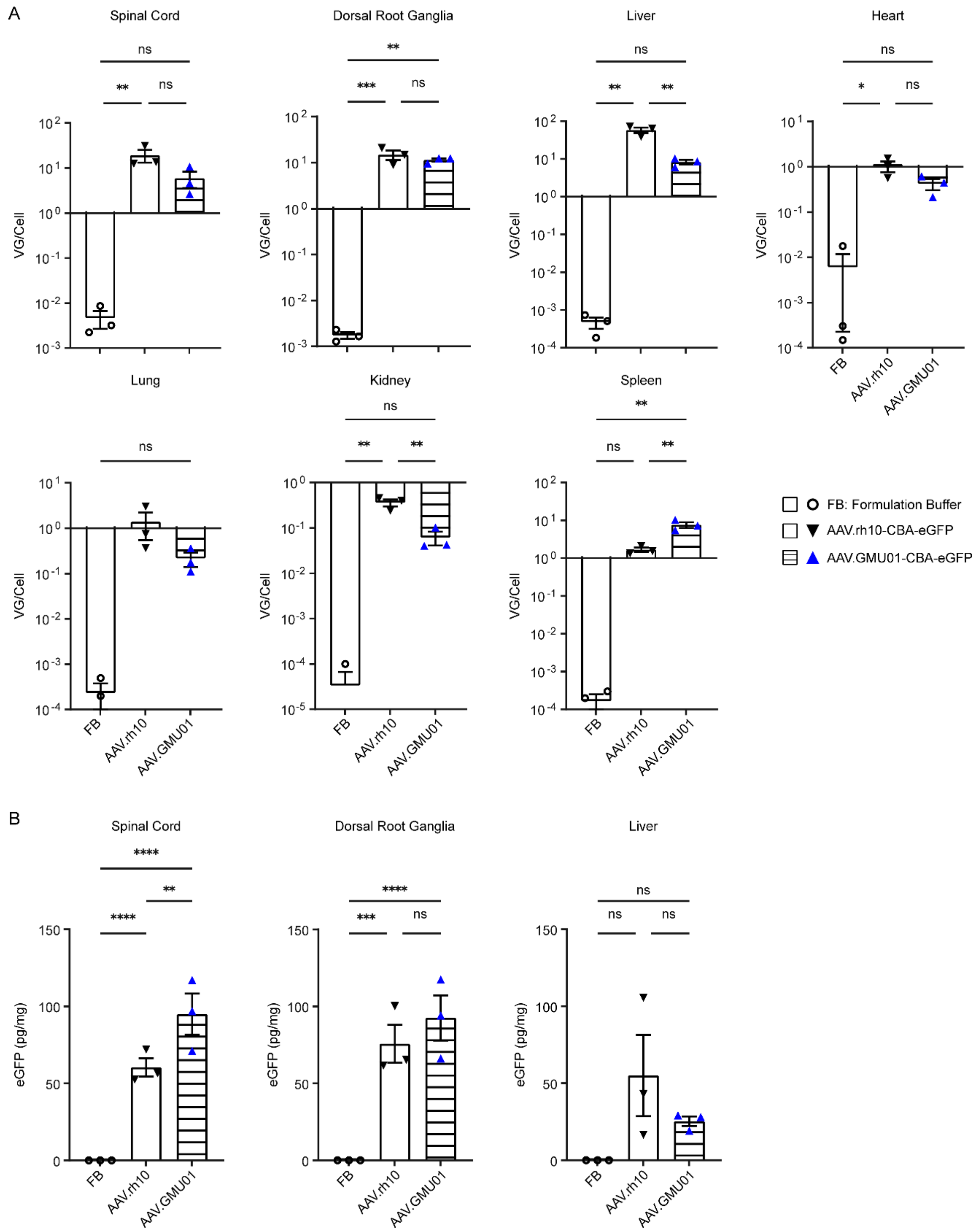

Supplementary Figure 1. The engineered capsid AAV.GMU01 shows higher transgene expression in the spinal cord and DRG of NHPs, compared to AAV.rh10. Cynomolgus monkeys (Male, Mauritian 2-3yr old, 2-3kg) seronegative for AAV.rh10 and AAV.GMU01 were dosed intra-thecal at the cervical level 1-2 junction using a ported intrathecal catheter inserted at the lumbar region. Animals were dosed in the Trendelenburg position. One dose of AAV.GMU01-CBA-eGFP, AAV.rh10-CBA-eGFP or formulation buffer was administered at 2.75e13VG/NHP (3.65e11VG/gm brain weight). Two weeks post-dosing, animals were euthanized, and samples were assessed for vector genome exposure and eGFP expression. Spinal cord and DRG from 7 segments along the spinal column. (A) AAV vector exposure by dPCR -(B) eGFP expression by ELISA.

Error bars represent mean with standard error. Two-way ANOVA with Tukey's multiple comparison test. $^{*}p<0.05$; $^{**}p<0.01$; $^{***}p<0.001$; $^{****}p<0.0001$.

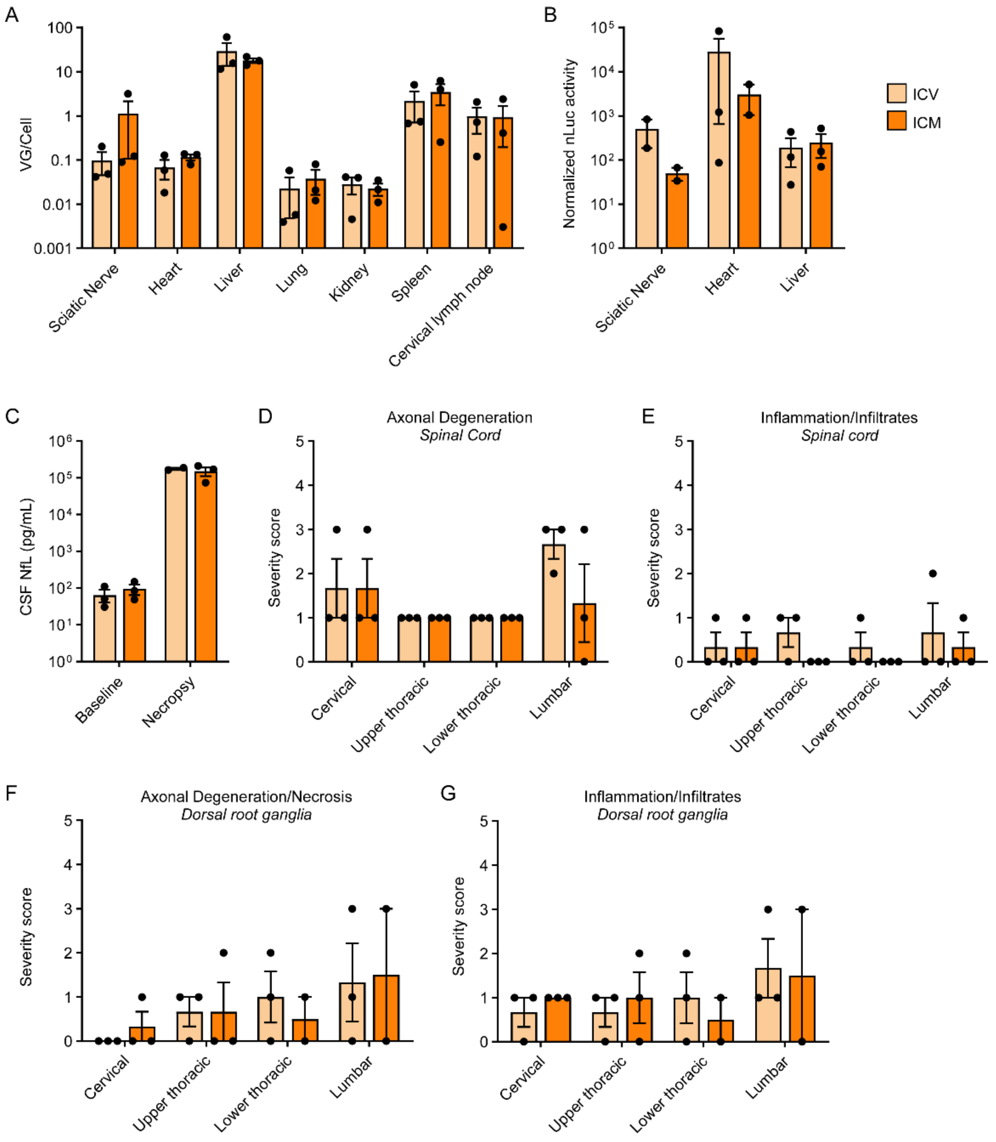

Supplementary Figure 2: AAV.GMU01 shows widespread vector biodistribution and transgene activity in peripheral tissues. Cynomolgus monkeys (Male, Vietnam, 2-3yr old, 2-3kg) seronegative for AAV.GMU01 were dosed with AAV.GMU01-CBA-NLuc-mCherry at 2.0e13VG/NHP (2.75e11 VG/gm brain weight) either by bilateral intracerebroventricular injection (ICV) or direct injection to the cisterna magna (ICM). Four weeks post-dosing, animals were euthanized, and tissues were flash-frozen from peripheral organs. (**A**) AAV vector exposure by bGH-dPCR, (**B**) nanoluciferease (nLuc) activity, normalized to total protein measured by bicinchoninic acid (BCA) assay. (**C**) Cerebrospinal fluid (CSF) samples were collected both prior to dosing ("prestudy") and at the four-week necropsy timepoint and were assayed by Quanterix Simoa to quantify neurofilament light chain (NfL), a measure of neuronal injury. (**D-G**) Histopathological findings in brain, spinal cord, and DRG; each data point represents the maximum severity of findings scored on 1-2 sections per animal. Severity scores refer to findings graded as 0= no findings, 1=minimal, 2=mild, 3=moderate, 4=marked, 5=severe. Error bars represent mean with standard error.

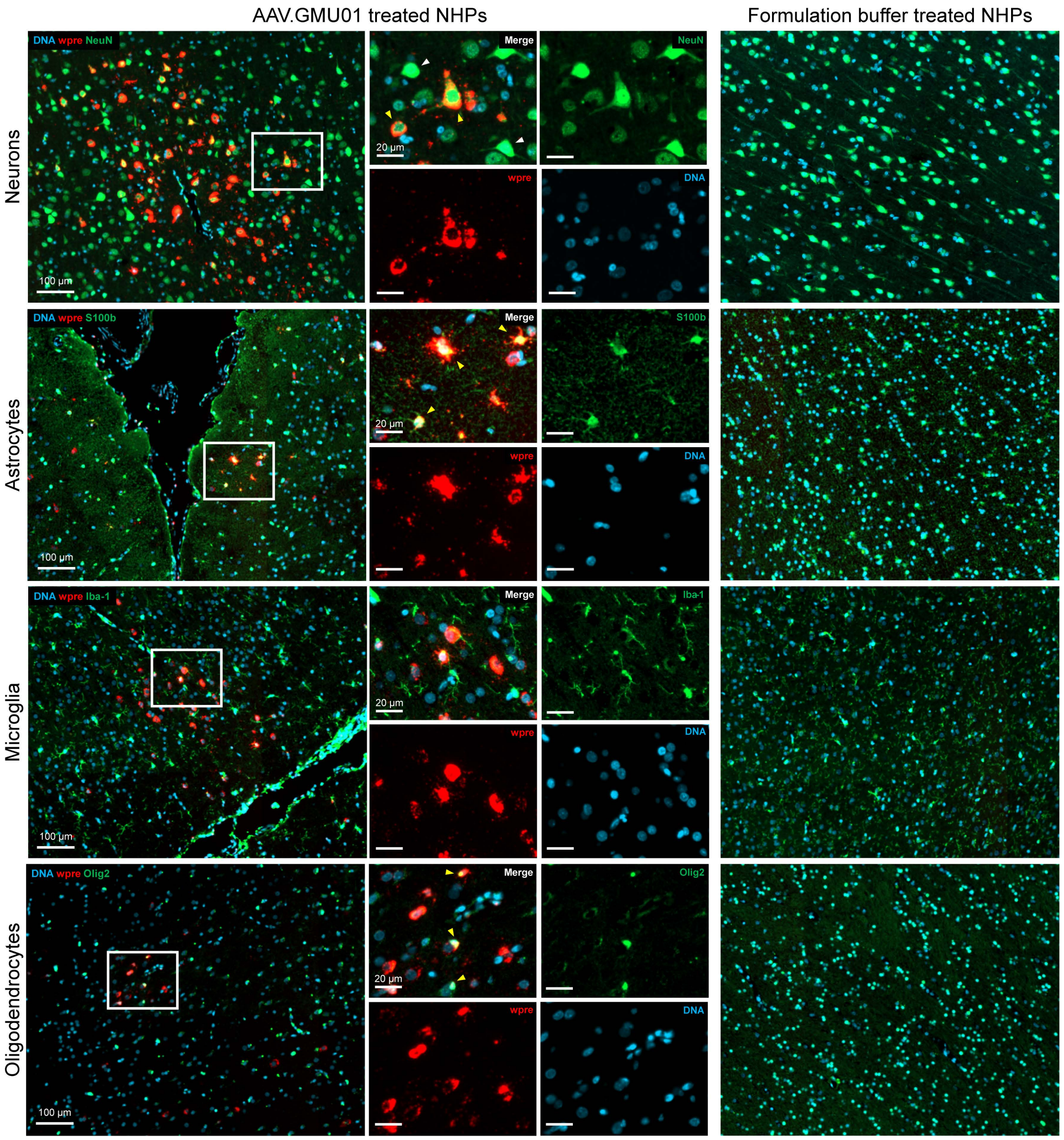

Supplementary Figure 3: AAV.GMU01 shows evidence of transducing neurons, astrocytes, microglia and oligodendrocytes in NHP brain. Representative images depicting co-detection of WPRE RNA and different cell type markers. Yellow arrowhead: transduced cell; White arrowhead: un-transduced cell. Staining and imaging details in Methods section.

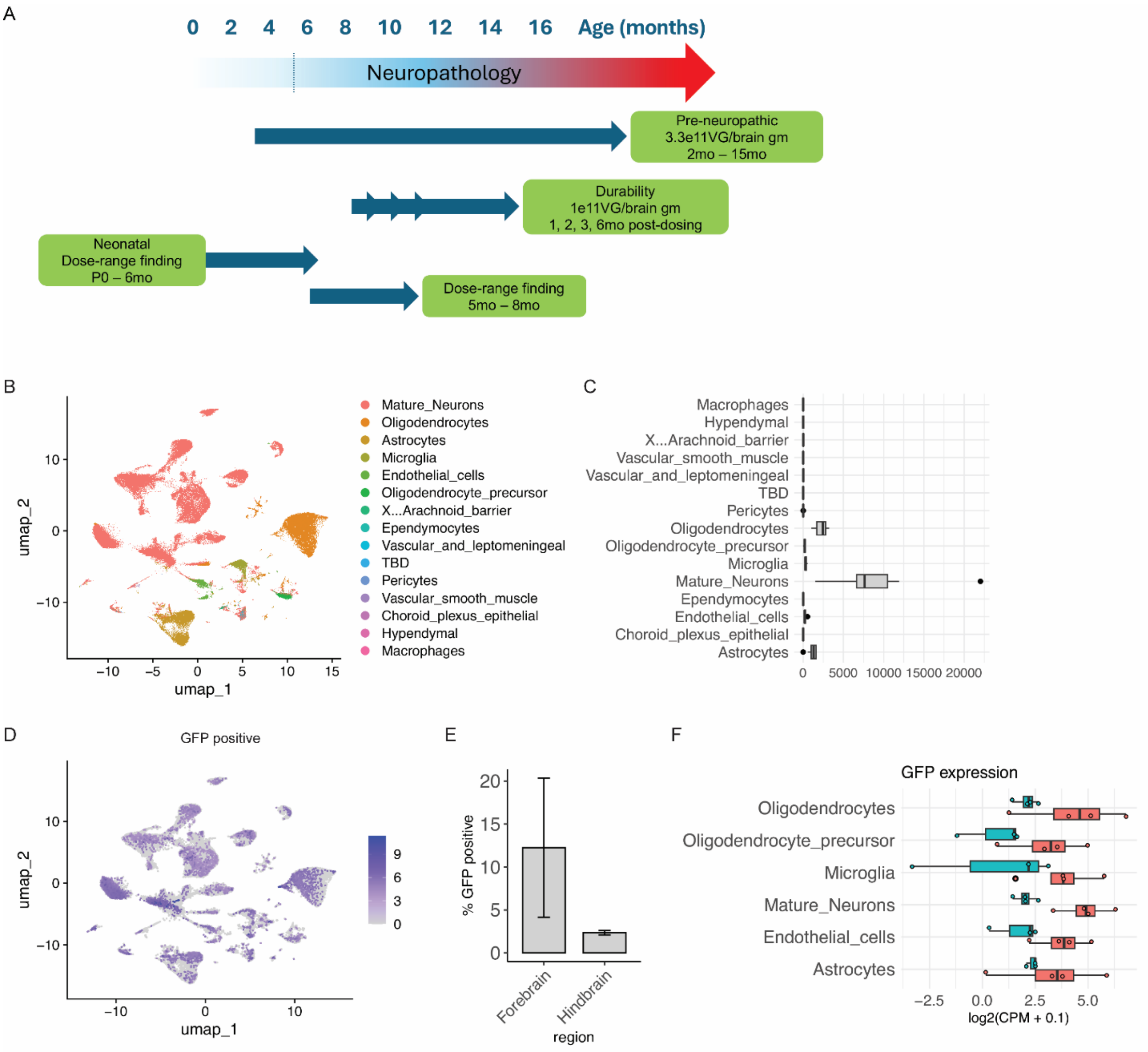

Supplementary Figure 4: Pharmacology studies in *Arsa* KO mice treated with AAV.GMU01-*ARSA* (A) Overview of pharmacology studies in *Arsa* KO mice treated with AAV.GMU01-*ARSA*. Single-cell RNA sequencing in mouse brain treated with AAV.GMU01-GFP (**B**) UMAP of cell states in mouse brains transfected with AAV1999. (**C**) Distribution of cell states in the data. Total cell frequencies (left) and proportions of cell states (right) are presented. (**D**) Feature plot of GFP positive cells on UMAP projection. (**E**) Percentage of GFP positive cells in the forebrain and hindbrain in different samples. Error bars represent standard error of the means between the samples. (**F**) Bar plots of GFP expression per cell states in the forebrain (red bars) and hindbrain (teal bars). Dots represent expression in different samples.

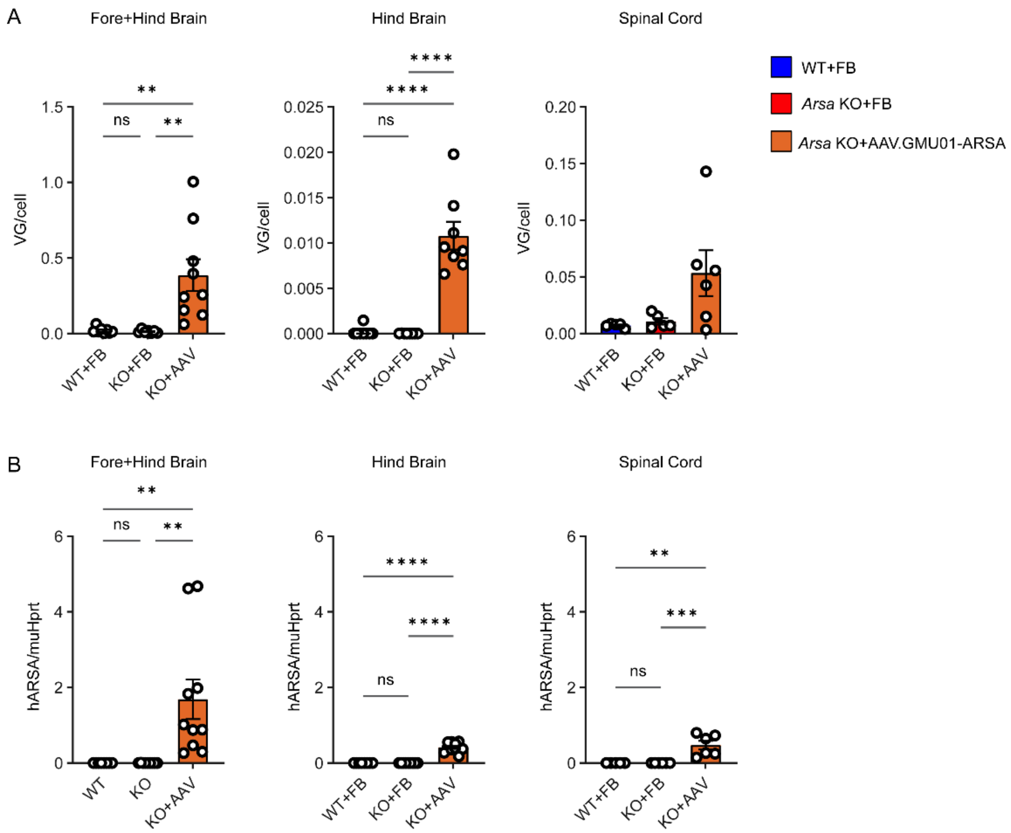

Supplementary Figure 5: *Arsa* KO Mice Treated with AAV.GMU01-*ARSA* show CNS-wide ARSA expression. Pre-neuronopathic *Arsa* KO mice and age-matched control animals were dosed with formulation buffer or AAV.GMU01-*ARSA* at 1.6e11VG/mouse (3.3e11VG/gram brain weight). Thirteen months post-dose, *Arsa* KO mice were euthanized, and brain and spinal cord samples collected. (**A**) Vector exposure by bGH-dPCR, (**B**) human *ARSA* mRNA levels by RT-dPCR. Error bars represent mean with standard error. One-way ANOVA with Tukey's multiple comparison test. *p<0.05; **p<0.01; ***p<0.001; ****p<0.0001. FB= Formulation Buffer.

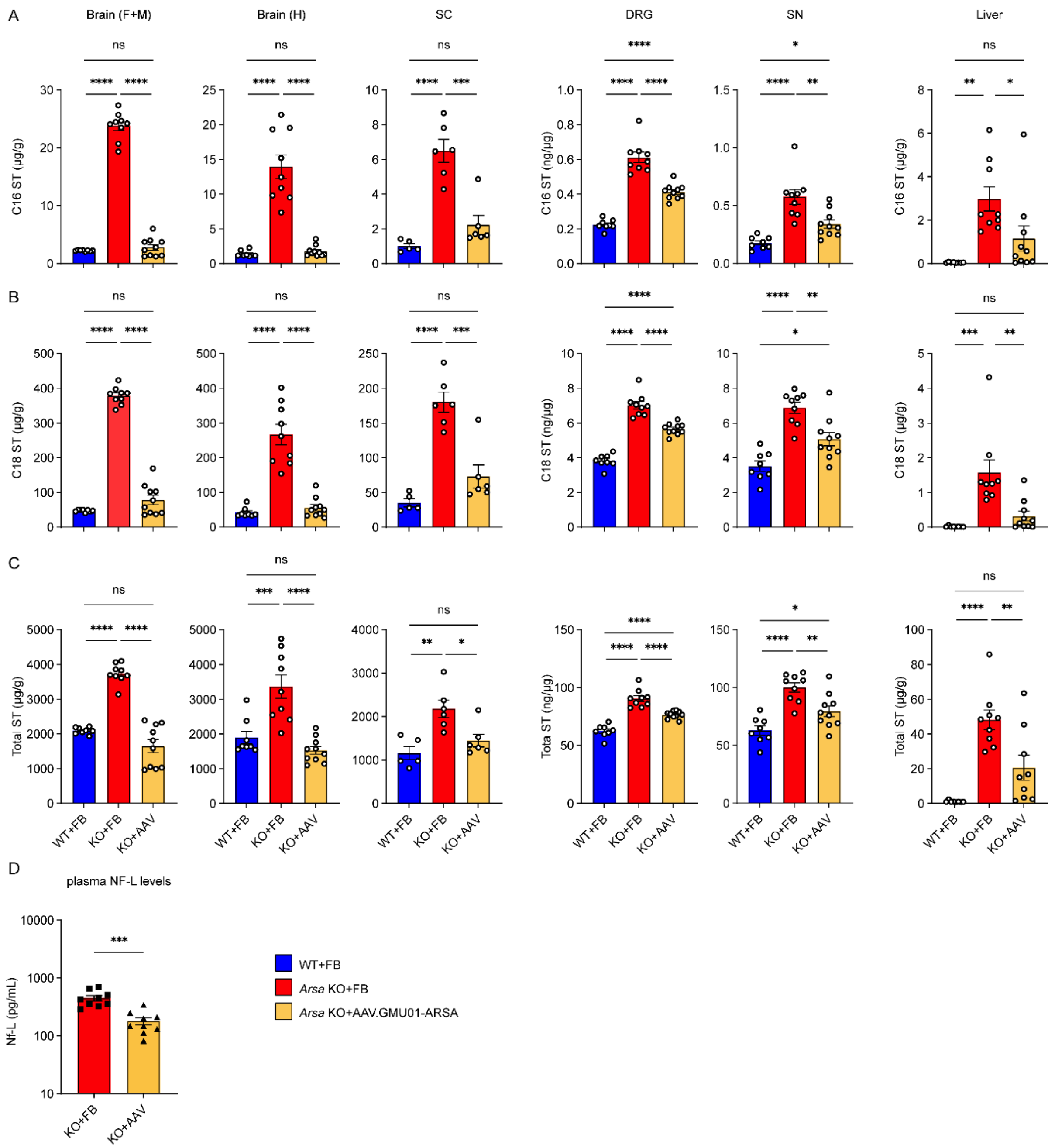

Supplementary Figure 6: Phenotypic reversal in *Arsa* KO mice treated with AAV.GMU01-*ARSA*. Pre-neuronopathic *Arsa* KO mice and age-matched control animals were dosed with formulation buffer or with AAV.GMU01-*ARSA* at 1.6e11VG/mouse (3.3e11VG/gram brain weight). Thirteen months post-dose, *Arsa* KO mice were euthanized and brain, spinal cord, DRG, sciatic nerve and liver samples collected. (**A-C**) Sulfatide levels were measured using liquid chromatography–mass spectrometry (LC-MS). Data normalized to tissue weight: converted from ng/mL (50 µL) to µg/g (ng/µg for DRG and SN). **(D)** Plasma samples were collected at necropsy and were assayed by Quanterix Simoa to quantify neurofilament light chain (NfL). Error bars represent mean with standard error. One-way ANOVA with Tukey's multiple comparison test. **p*<0.05; ***p*<0.01; ****p*<0.001; *****p*<0.0001. FB= Formulation Buffer, sulfatide= ST.

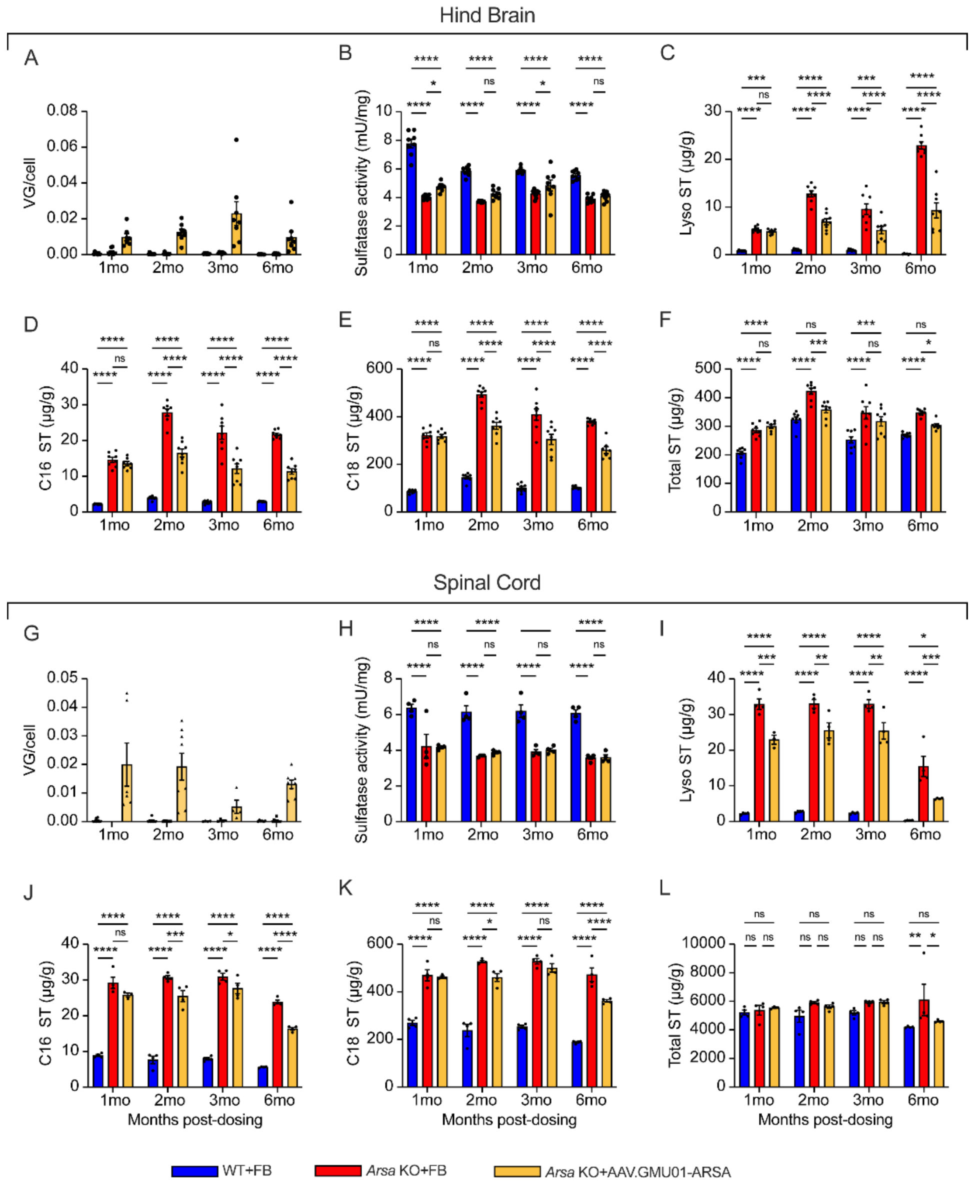

Supplementary Figure 7: ARSA expression and function is persistent over time. Early-neuronopathic *Arsa* KO mice (6mo at dosing) and age-matched control animals were dosed with formulation buffer or AAV.GMU01-*ARSA* at 5e10VG/mouse (1.0e11VG/gram brain weight). One, two, three and six months post-dose, mice were euthanized, and samples collected. (**A-F**) Hind brain samples, (**G-L**) Spinal Cord. (**A, G**) Vector exposure by bGH-dPCR normalized to the *Rab1a* gene (intronic region). (**B, H**) ARSA-mediated sulfatase activity was measured using the Sulfatase Activity Assay Kit, data normalized to total protein measured by BCA assay. (**C, I**) Lyso-sulfatide, (**D, J**) C16-sulfatide isoform, (**E, K**) C18-sulfatide isoform and (**F, L**) Total sulfatide levels were measured using LC-MS. Data normalized to tissue weight: converted from ng/mL (50 µL) to µg/g. Error bars represent mean with standard error. Two-way ANOVA with Tukey's multiple comparison test. *p<0.05; **p<0.01; ***p<0.001; ****p<0.0001. FB= Formulation Buffer, sulfatide= ST.

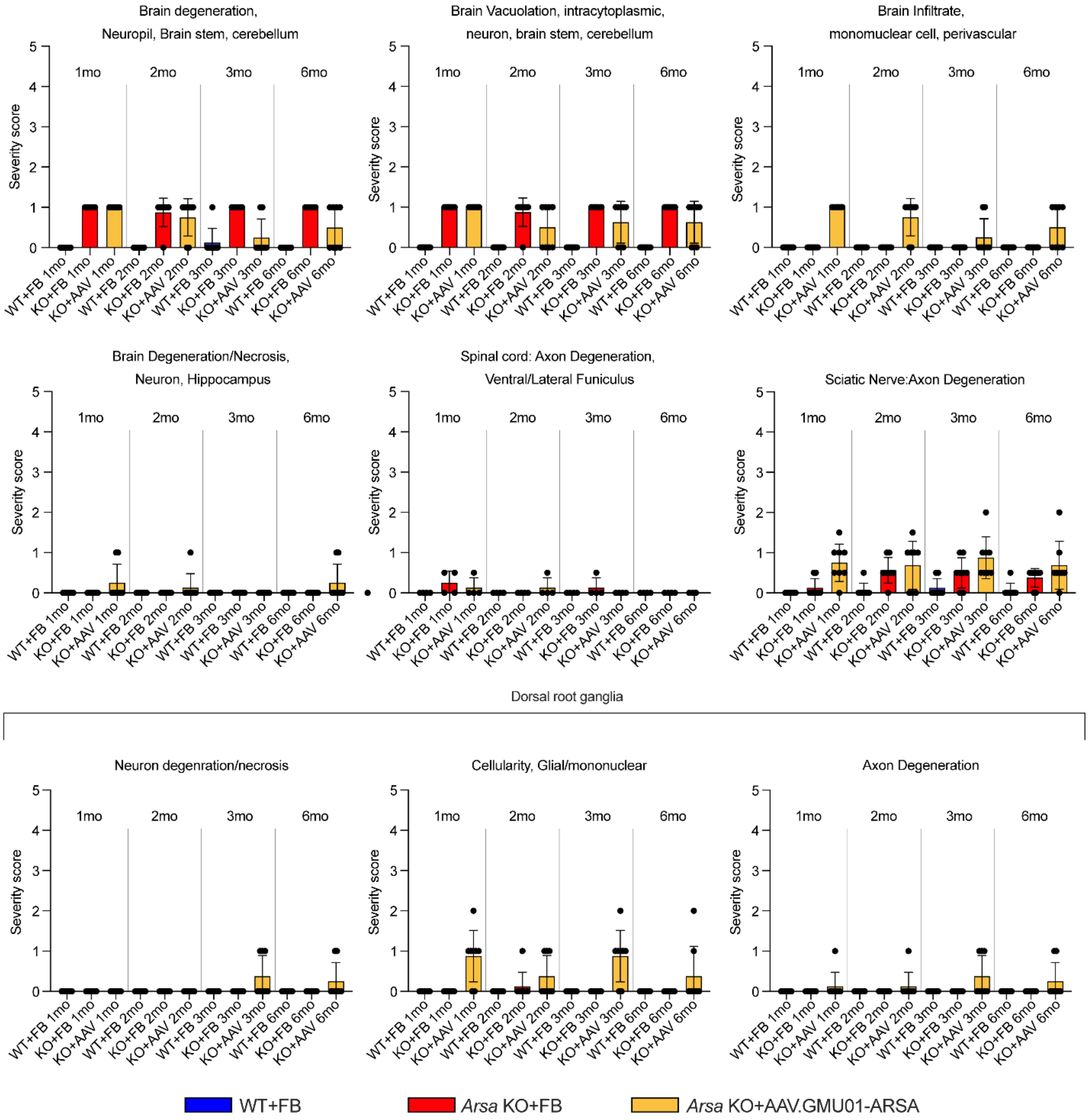

Supplementary Figure 8: AAV.GMU01-*ARSA* treated *Arsa* KO mice show up to mild findings. Histopathological findings in brain, spinal cord, sciatic nerve and DRG in *Arsa* KO treated at 6 months (early-neuronopathic); each data point represents the maximum severity of findings scored on 1-2 sections per animal. Severity scores refer to findings graded as 0= no findings, 1=minimal, 2=mild, 3=moderate, 4=marked, 5=severe. Error bars represent mean with standard error. FB= Formulation Buffer.

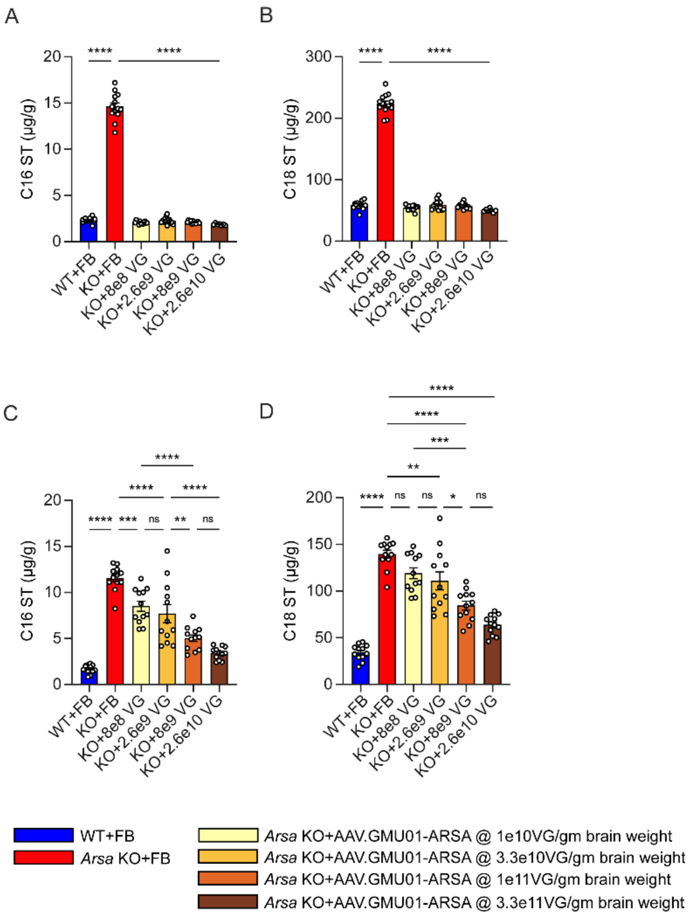

Supplementary Figure 9. AAV.GMU01-*ARSA* treated results in dose-dependent sulfatide clearance in *Arsa* KO mice. (**A-B**) Neonatal *Arsa* KO mice (P0 at dosing) and age-matched control animals were dosed with formulation buffer or AAV.GMU01-*ARSA* at the given doses. Six months post-dose, mice were euthanized and samples collected. (**C-D**) Early-neuronopathic *Arsa* KO mice (6mo at dosing) and age-matched control animals were dosed with AAV.GMU01-*ARSA* at noted doses. Three months post-dose, mice were euthanized, and samples collected. (**A, C**) C16 sulfatide isoform and (**B, D**) C18 sulfatide isoform levels were measured using LC-MS. Data normalized to tissue weight: converted from ng/mL (50 µL) to µg/g. Data normalized to tissue weight: converted from ng/mL (50 µL) to µg/g. Error bars represent mean with standard error. Two-way ANOVA with Tukey's multiple comparison test. *p<0.05; **p<0.01; ***p<0.001; ****p<0.0001. FB= Formulation Buffer, sulfatide= ST; gbw= grams per brain weight.

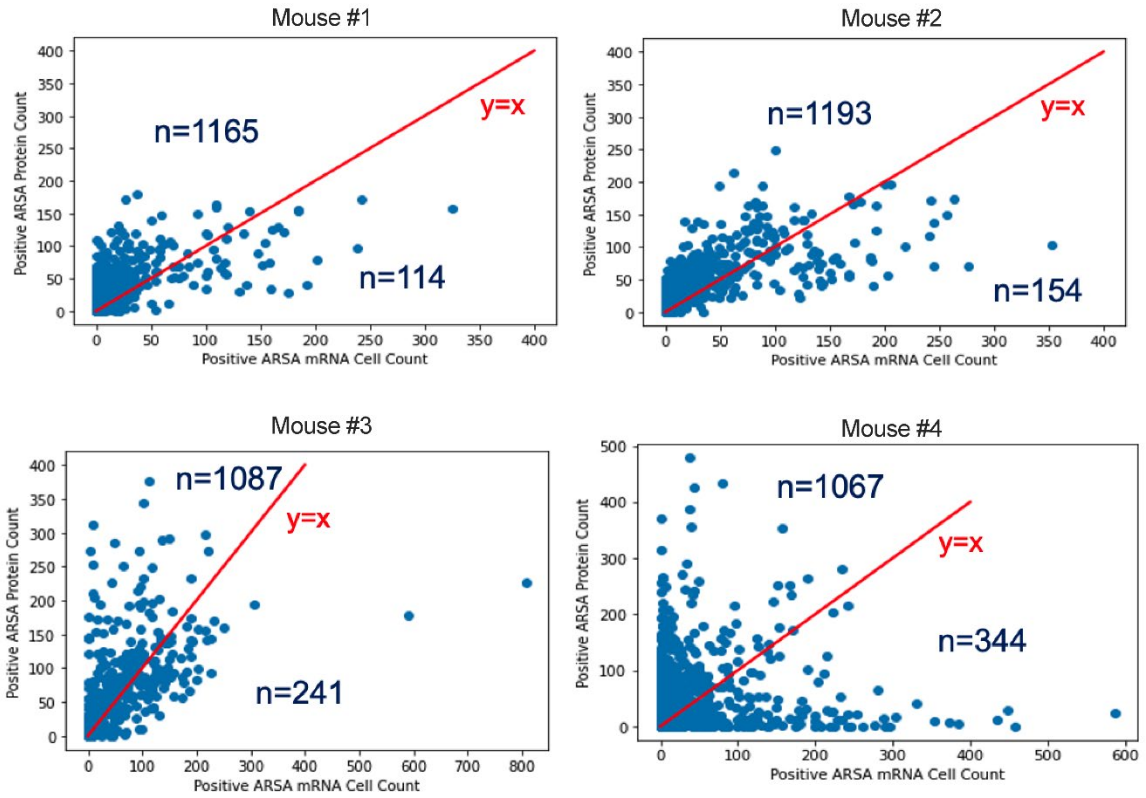

Supplementary Figure 10: AAV.rh10-CBA-ARSA-WPRE treated *Arsa* KO mice show evidence of ARSA protein cross-correction. Late-stage (13 month) *Arsa* KO mice were dosed with AAV.rh10-CBA-ARSA-WPRE. Three months post-dosing, ARSA-mRNA *in situ* hybridization (ISH) and ARSA-protein immunohistochemistry (IHC) were performed on matched sagittal brain hemi-sections. The sections were imaged and analyzed for signal overlay. Adjacent sagittal sections from mouse brain were treated for WPRE ISH or ARSA IHC with DAPI staining for nuclei. The sagittal sections were then analyzed as individual 1024x1024 pixel tiles. In each tile, ISH positive cells and IHC positive cells were determined by thresholding parameters empirically determined across the entire sagittal images. ISH+ cell count vs IHC+ cell count from each tile is plotted as a scatter plot with y=x line shown in red. Tiles above the y=x line indicates cross-corrected cells.

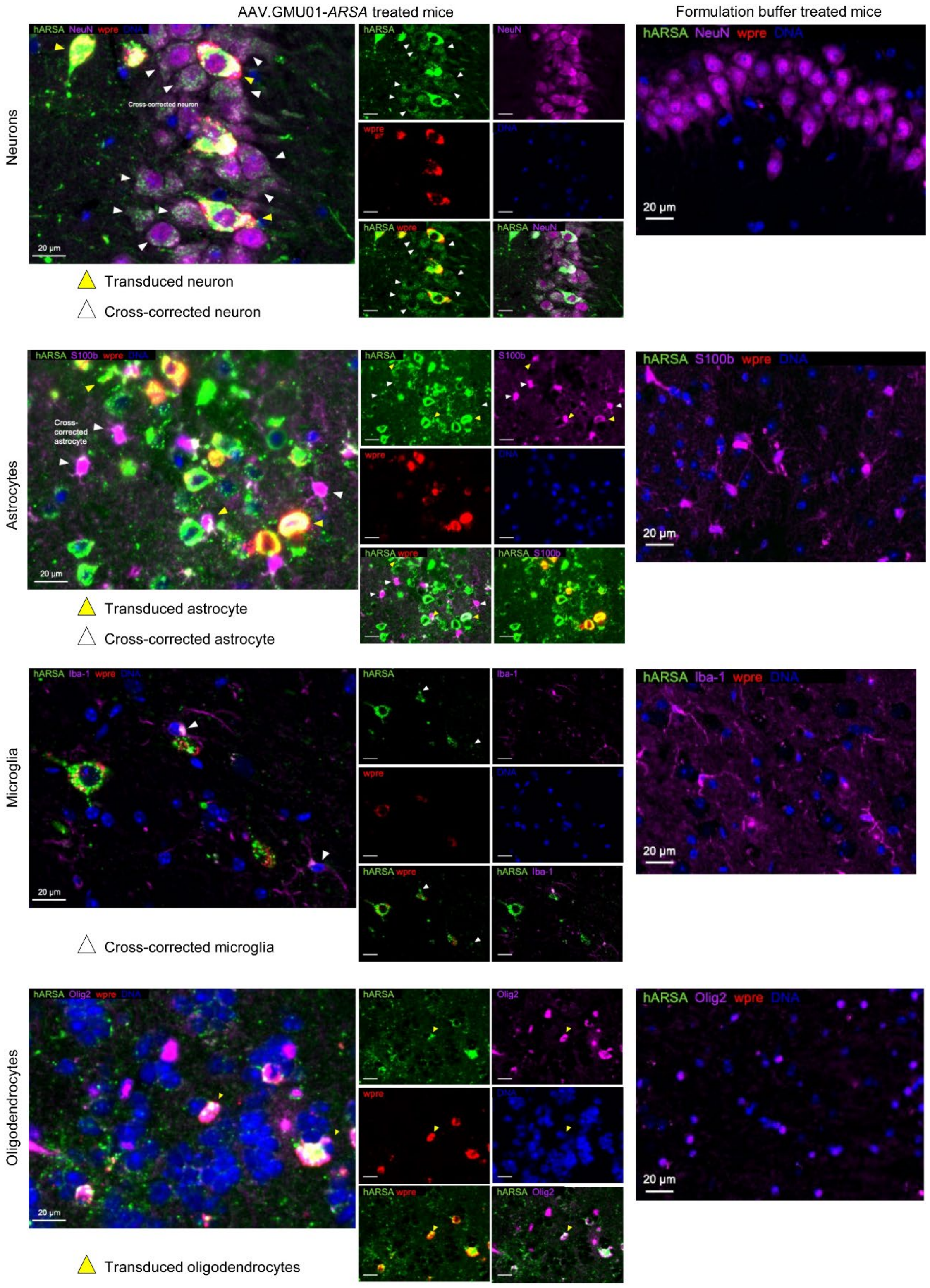

Supplementary Figure 11: Evidence of ARSA protein cross-correction in *Arsa* KO mice upon treatment with AAV.GMU01-*ARSA*. Representative images depicting co-detection of WPRE RNA, ARSA protein and different cell type markers. Yellow arrowhead: transduced cell (positive for wpre and cell-type marker); White arrowhead: cross-corrected cell (positive for ARSA and cell-type marker but negative for wpre). Staining and imaging details in Methods section.

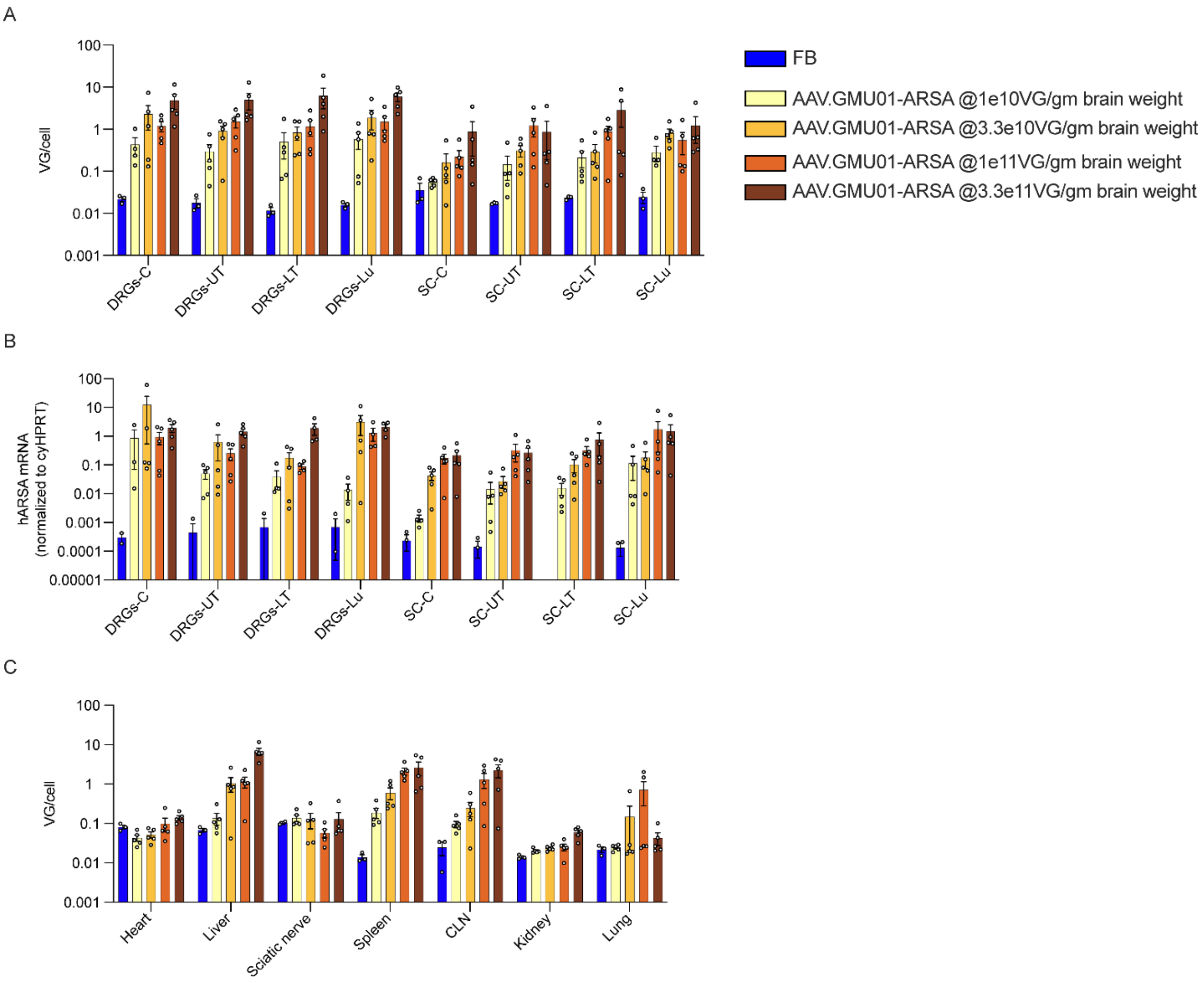

Supplementary Figure 12: Widespread dose-dependent vector biodistribution and ARSA expression in spinal cord, DRGs and peripheral tissues. Eight spinal cord segments with adjacent DRGs, 2 each from cervical, upper thoracic, lower thoracic and lumbar were flash frozen and DNA/RNA isolated. (A, C) Digital PCR (dPCR) was performed to quantify AAV.GMU01 vector concentration and normalized to the *TUBB1* gene intron. (B) RT-dPCR was performed to quantify *ARSA* mRNA expression, normalized to endogenous *HPRT* gene. Each data point represents VG/cell exposure or normalized *ARSA* expression for that sample, averaged across all NHPs in that group. Error bars represent mean with standard error. FB= Formulation Buffer. C=cervical, UT=upper thoracic; LT=lower thoracic; Lu=lumbar; CLN=cervical lymph node

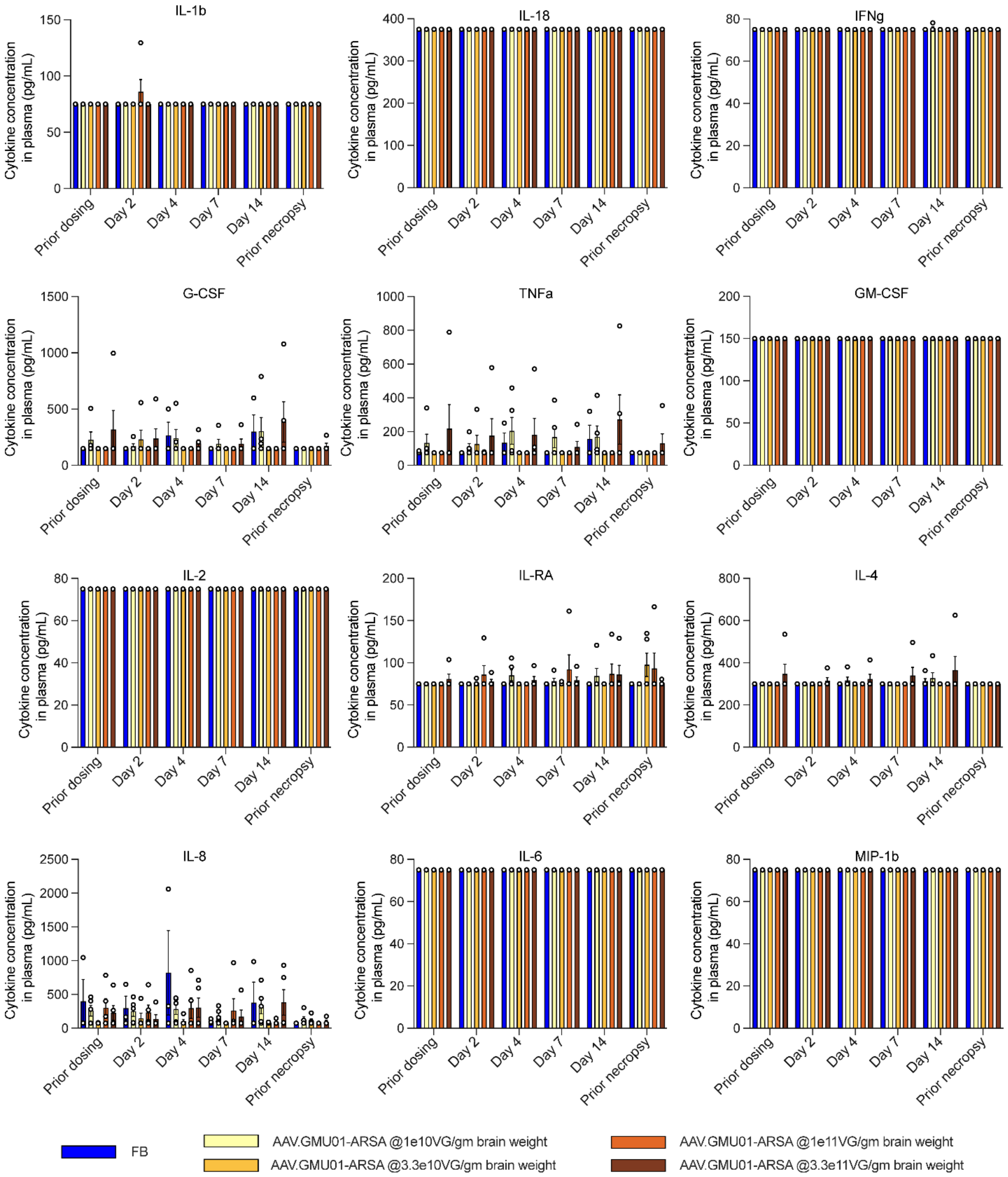

Supplementary Figure 13: ICM infusion was well tolerated and did not trigger innate immune responses. Plasma was isolated pre-dose and at Days 2, -4, 7, 14 post-dose and at necropsy. The Luminex assay was used to determine the concentration of IL-1b, IL-1RA, IL-6, IL-10, IL12/23 (p40), IL-15, IL-18, IFN-g, TNF-a, G-CSF, MCP-1, MIP-1b, GM-CSF, IL-2, IL-4, IL-5, IL-8, IL-13 and IL-17A. Each data point represented cytokine concentration in that sample, averaged across all NHPs in that group. Error bars represent mean with standard error.

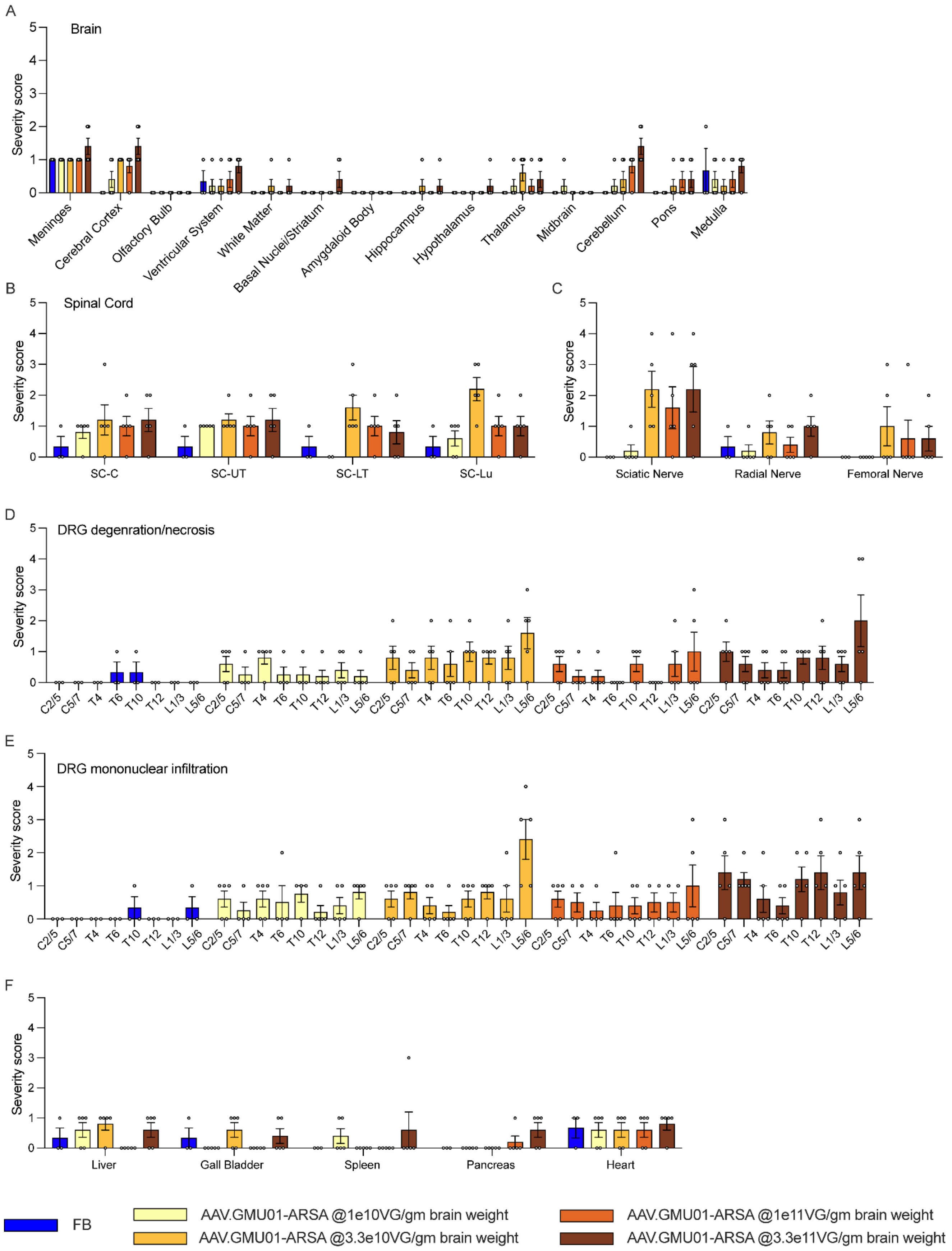

Supplementary Figure 14: ICM infusion was well tolerated in NHPs. Histopathological findings in brain, spinal cord, and DRG; each data point represents the maximum severity of findings scored on 1-2 sections per animal. Refer to Supplementary Table 6 for study design. Severity scores refer to findings graded as 0= no

findings, 1=minimal, 2=mild, 3=moderate, 4=marked, 5=severe. Error bars represent mean with standard error. C=cervical, T=thoracic; L=lumbar (numbers represent segments reviewed by pathologist)

SUPPLEMENTARY TABLES

Supplementary Table 1: Study design evaluating AAV.GMU01 and AAV.rh10 vector biodistribution and transgene expression in CNS

Group	# of Animals	Test Article	Dose (vg)	Dose (vg/gm brain weight)	RoA	Dosing paradigm	Necropsy
**1**	3 (M)	Formulation Buffer	N/A	N/A	Intrathecal Catheter (C1-C2)	2.5ml>6hr >2.5ml	29 days
**2**	3 (M)	AAV.rh10-CBA-eGFP	2.75E+13	2.65E+11			16 days
**3**	3 (M)	AAV.GMU01-eGFP	2.75E+13	2.65E+11			18 days

Supplementary Table 2: Study design comparing AAV.GMU01 biodistribution and transgene activity by ICV and ICM administration routes

Group	# of Animals	ROA	Description	Animal position	Dose	Volume	Rate	Necropsy
**1**	3 (M)	Bilateral ICV	Clearpoint, Sequential	Sternal recumbent followed by Trendelenburg	2.0e13 VG/ NHP	1ml per hemisphere	0.125 ml/min	28±1 days
**2**	3 (M)	Direct ICM	CM puncture with needle	Trendelenburg		2ml		

Supplementary Table 3: Long-term Pharmacology, Efficacy and Durability Study in *Arsa* KO mice (2mo-15mo)

Group	Number/sex	Genotype	Test article	Dose	Dosing regimen	Time Points
**1**	12 (6M, 6F)	WT	Formulation buffer	N/A	Bi-lateral ICV, 4 uL each side	Dosing age: 2 mo Necropsy age: 15 mo (13 mo in-life)
**2**	12 (6M, 6F)	$ARSA^{-/-}$	Formulation buffer	N/A		
**3**	12 (6M, 6F)	$ARSA^{-/-}$	AAV.GMU01-*ARSA*	1.6e11VG (3.3e11VG/gram brain weight)		

Supplementary Table 4: Longitudinal Pharmacology, Efficacy and Durability Study in *Arsa* KO Mice

Group	Number and Sex	Genotype	Test Article	Dose	Dosing Regimen	Time Points
**1**	32 (16M, 16F)	WT	Formulation buffer	N/A	Bi-lateral ICV, 4ul each side	Dosing age: 6 mo<br>Necropsy age: 7 mo (1 mo in-life), 8 mo (2 mo in-life), 9 mo (3 mo in-life), 12 mo (6 mo in-life)
**2**	32 (16M, 16F)	$Arsa^{-/-}$	Formulation buffer	N/A		
**3**	32 (16M, 16F)	$Arsa^{-/-}$	AAV.GMU01-*ARSA*	5e10VG (1e11VG/gram brain weight)		

Supplementary Table 5: Dose-dependent pharmacology studies in *Arsa* KO Mice

**Pharmacology and dose optimization study in P0 neonatal mice**						
**Group**	**Number and Sex**	**Genotype**	**Test Article**	**Dose**	**Dosing Regimen**	**Time Points**
**1**	12 (6M, 6F)	WT	Formulation buffer	N/A	Bi-lateral ICV, 3ul each side	Dosing age: P0<br>Necrospy: 6 mo<br>(6mo in-life)
**2**	12 (6M, 6F)	*Arsa*$^{-/-}$	Formulation buffer	N/A		
**3**	12 (6M, 6F)		AAV.GMU01-*ARSA*	2.64e10VG (3.3e11VG/gram brain weight)		
**4**	12 (6M, 6F)			8.0e9VG (1.0e11VG/gram brain weight)		
**5**	12 (6M, 6F)			2.64e9VG (3.3e10VG/gram brain weight)		
**6**	12 (6M, 6F)			8.0e8VG (1.0e10VG/gram brain weight)		
**Pharmacology and dose optimization study in early-neuronopathic mice**						
**Group**	**Number and Sex**	**Genotype**	**Test Article**	**Dose**	**Dosing Regimen**	**Time Points, Sample Collection, Analysis**
**1**	12 (6M, 6F)	WT	Formulation buffer	N/A	Bi-lateral ICV, 5ul each side	Dosing age: 6 mo<br>Necrospy: 9 mo<br>(3mo in-life)
**2**	12 (6M, 6F)	*Arsa*$^{-/-}$	Formulation buffer	N/A		
**3**	12 (6M, 6F)		AAV.GMU01-*ARSA*	1.6e11VG (3.3e11VG/gram brain weight)		
**4**	12 (6M, 6F)			- 5.0e10VG (1.0e11VG/gram brain weight)		
**5**	12 (6M, 6F)			- 1.6e10VG (3.3e10VG/gram brain weight)		
**6**	12 (6M, 6F)			- 5.0e9VG (1.0e10VG/gram brain weight)		

Supplementary Table 6: NHP dose-ranging and tolerability study

Group	Test Article	Dose/gm Brain Weight	Dose/Animal	Dosing Regimen	# of Animals	Time Points, Sample Collection
**1**	AAV.GMU01-*ARSA*	1e10 VG	7.5e11 VG	RoA: ICM with animal placed in Trendelenburg position<br><br>Doing parameters: 2.5ml @0.125mL/min with 250ul flush	5 (M/F)	In-life: 35 days<br><br>CSF (pre-study, and at necropsy)<br><br>Plasma: pre-study, days post-dose: 2, 4, 7, 14 and at necropsy<br><br>Neurological/behavioral assessment (pre-dose,<br>7 days and 5 weeks post-dose)
**2**		3.3e10 VG	2.5e12 VG		5 (M/F)	
**3**		1e11 VG	7.5e12 VG		5 (M/F)	
**4**		3.3e11 VG	2.5e13 VG		5 (M/F)	
**5**	Formulation Buffer	n/a	n/a		3 (M/F)	

Supplementary Table 7. Neurological and Behavioral Battery performed pre-dosing, on day 7 post-dose and at necropsy (Week 5 Post-dose).

General observations	Attitudinal Posture Reactions	Spinal Segmental Reflex	Cranial Nerve Function
Mental status	Tonic neck	Perineal Reflex	Pathologic Nystagmus
Head posture	Hopping	Withdrawal reflex	Facial sensory Exam
Motor activity	Visual placing	Cross extensor	Olfactory Nerve Exam
Coordination	Tactile placing	Patellar reflex	Jaw tone
Stance	Postural reaction	Babinski reflex	Gag reflex
Gait		Chaddock reflex	Facial symmetry
Circling			Cochlear Nerve Exam
			Tongue Exam
			Menace Response
			Pupilliary light reflex (PLR)
			PLR Consensual
			Pupil Size
			Pupil symmetry
			Ocular position
			Ocular motility
			Oculovestibular reflex
			Palpebral reflex

Supplementary Table 8. Reagents for fluorescent RNA *in situ* hybridization

Kits	Vendor	Catalog No.
RNAscope^{TM} LS Multiplex Fluorescent	ACD	322800
Wpre-O1 probe	ACD	450268
Bond Polymer Refine Detection Kit	Leica Biosystem	DS9800
BOND Research Detection System	Leica Biosystem	DS9455
TSA Vivid 650	R&D Systems	75271KIT
RNAscope^{TM} LS Multiplex Fluorescent	ACD	322800
Wpre probe	ACD	410058

Supplementary Table 9. Antibodies for *in situ* hybridization.

Primary antibody			
**Target**	**Host**	**Vendor**	**Catalog No.**
Human arylsulfatase A/ARSA, pAb	Goat	R&D Systems	AF2485
Anti-NeuN antibody [EPR12763], mAb	Rabbit	Abcam	ab177487
Iba1/AIF-1 (E4O4W) XP, mAb	Rabbit	Cell Signaling Technology	17198S
S100B (E7C3A)	Rabbit	Cell Signaling Technology	42397
Anti-mCherry [1C51]	Mouse	Millipore Sigma	MAB131873
Anti-NeuN antibody [EPR12763], mAb	Rabbit	Abcam	ab177487
Iba1/AIF-1 (E4O4W) XP, mAb	Rabbit	Cell Signaling Technology	17198S
S100B (E7C3A)	Rabbit	Cell Signaling Technology	42397
Anti-Olig2 [EPR2673]	Rabbit	Abcam	ab109186
**Secondary antibody**			
**Target**	**Host**	**Vendor**	**Catalog No.**
Anti-Rabbit IgG – Alexa Fluor 488	DK	Thermo	AF32790
Anti-Goat IgG – Alexa Fluor 555	DK	Thermo	AF32816
Anti-Mouse IgG – Alexa Fluor 555	DK	Thermo	AF32773
